# Global responses to the COVID-19 pandemic by recreational anglers: considerations for developing more resilient and sustainable fisheries

**DOI:** 10.1007/s11160-023-09784-5

**Published:** 2023-05-30

**Authors:** J. Robert Britton, Adrian C. Pinder, Josep Alós, Robert Arlinghaus, Andy J. Danylchuk, Wendy Edwards, Kátia M. F. Freire, Casper Gundelund, Kieran Hyder, Ivan Jarić, Robert Lennox, Wolf-Christian Lewin, Abigail J. Lynch, Stephen R. Midway, Warren M. Potts, Karina L. Ryan, Christian Skov, Harry V. Strehlow, Sean R. Tracey, Jun-ichi Tsuboi, Paul A. Venturelli, Jessica L. Weir, Marc Simon Weltersbach, Steven J. Cooke

**Affiliations:** 1 Department of Life and Environmental Sciences, Faculty of Science and Technology, Bournemouth University, Poole, BH12 5BB UK; 2 Instituto Mediterráneo de Estudios Avanzados, IMEDEA (CSIC–UIB), Esporles, Spain; 3 Department of Fish Biology, Fisheries and Aquaculture, Leibniz Institute of Freshwater Ecology and Inland Fisheries, Müggelseedamm 310, 12587 Berlin, Germany; 4 Division of Integrative Fisheries Management, Faculty of Life Sciences, Humboldt-Univesität zu Berlin, Invalidenstrasse 42, 10115 Berlin, Germany; 5 Department of Environmental Conservation, University of Massachusetts Amherst, Amherst, MA 01003 USA; 6 Centre for Environment, Fisheries and Aquaculture Science (Cefas), Pakefield Road, Lowestoft, NR33 0HT Suffolk UK; 7 Department of Fisheries Engineering and Aquaculture, Universidade Federal de Sergipe, Cidade Universitária Prof. José Aloísio de Campos, Rua Mal. Rondon S/N, Jardim Rosa Elze São Cristóvão, Sergipe CEP 49100-000 Brazil; 8 Section of Freshwater Fisheries and Ecology, Technical University of Denmark, DTU Aqua, 8600 Silkeborg, Denmark; 9 School of Environmental Sciences, University of East Anglia, Norwich Research Park, Norwich, NR4 7TJ Norfolk UK; 10 Biology Centre of the Czech Academy of Sciences, Institute of Hydrobiology, Na Sádkách 702/7, 37005 České Budějovice, Czech Republic; 11 Université Paris-Saclay, CNRS, AgroParisTech, Ecologie Systématique Evolution, 12 Rue 128, 91190 Gif-Sur-Yvette, France; 12 Norwegian Institute for Nature Research and at the Laboratory for Freshwater Ecology, Oslo, Norway; 13 Thünen Institute of Baltic Sea Fisheries, Alter Hafen Süd 2, 18069 Rostock, Germany; 14 U.S. Geological Survey, National Climate Adaptation Science Center, 12201 Sunrise Valley Drive MS 516, Reston, VA 20192 USA; 15 Department of Oceanography and Coastal Sciences, Louisiana State University, Baton Rouge, LA 70803 USA; 16 Department of Ichthyology and Fisheries Science, Rhodes University, P.O. Box 94, Makhanda, 6140 South Africa; 17 Department of Primary Industries and Regional Development, Western Australian Fisheries and Marine Research Laboratories, 39 Northside Drive, Hillarys, WA 6025 Australia; 18 Institute for Marine and Antarctic Studies, University of Tasmania, Private Bag 49, Hobart7001, TAS Australia; 19 Research Center for Freshwater Fisheries, Japan Fish Res and Education Agency, Nikko, 321-1661 Japan; 20 Department of Biology, Ball State University, Muncie, IN 47304 USA; 21 Fish Ecology and Conservation Physiology Laboratory, Department of Biology and Institute of Environmental and Interdisciplinary Science, Carleton University, 1125 Colonel By Dr., Ottawa, ON K1S 5B6 Canada; 22 Centre For Marine Socioecology, University of Tasmania, Private Bag 49, Hobart7001, TAS Australia

**Keywords:** Angling effort, Angling licence, Angler demographics, Culturomics, COVID-19 lockdow﻿n

## Abstract

The global COVID-19 pandemic resulted in many jurisdictions implementing orders restricting the movements of people to inhibit virus transmission, with recreational angling often either not permitted or access to fisheries and/or related infrastructure being prevented. Following the lifting of restrictions, initial angler surveys and licence sales suggested increased participation and effort, and altered angler demographics, but with evidence remaining limited. Here, we overcome this evidence gap by identifying temporal changes in angling interest, licence sales, and angling effort in world regions by comparing data in the ‘pre-pandemic’ (up to and including 2019); ‘acute pandemic’ (2020) and ‘COVID-acclimated’ (2021) periods. We then identified how changes can inform the development of more resilient and sustainable recreational fisheries. Interest in angling (measured here as angling-related internet search term volumes) increased substantially in all regions during 2020. Patterns in licence sales revealed marked increases in some countries during 2020 but not in others. Where licence sales increased, this was rarely sustained in 2021; where there were declines, these related to fewer tourist anglers due to movement restrictions. Data from most countries indicated a younger demographic of people who participated in angling in 2020, including in urban areas, but this was not sustained in 2021. These short-lived changes in recreational angling indicate efforts to retain younger anglers could increase overall participation levels, where efforts can target education in appropriate angling practices and create more urban angling opportunities. These efforts would then provide recreational fisheries with greater resilience to cope with future global crises, including facilitating the ability of people to access angling opportunities during periods of high societal stress.

**Supplementary Information:**

The online version contains supplementary material available at 10.1007/s11160-023-09784-5.

## Introduction

Recreational fishing is a highly popular leisure activity, with approximately 10% of the global population participating (Arlinghaus and Cooke 2009; Arlinghaus et al. 2019). Rod and line fishing (i.e. angling) is the most common form of recreational fishing, but other methods including spear fishing are also widespread. Motivations for recreational angling are diverse, but can be psychological (e.g., emotional benefits of being in the outdoors), personal challenge-related (e.g. trying to catch a ‘trophy’ sized fish), social (e.g. interacting with other anglers), competitive (e.g. tournament fishing) and nutritional (capturing fish for food) (Griffiths et al. 2017; Cooke et al. 2018; Nolan et al. 2019). Recreational angling generally makes important contributions to local and national economies (Parkkila et al. 2010). For example, recreational angling activities have been estimated to generate up to US$1.5B per annum in the Laurentian Great Lakes region (Lynch et al. 2016). In Europe, marine recreational fishers spend €5.9B each year (Hyder et al. 2018), generating a total economic impact of €10.5B (Hyder et al. 2017). Licence and permit sales also generate revenues that regulatory authorities often use to finance recreational angling and fish conservation programmes (Tufts et al. ﻿^2015^).

The global COVID-19 pandemic resulted in many nations and jurisdictions implementing lockdown orders to limit the movements of people and inhibit virus transmission. As a result, over a third of the global population was under some form of restrictions in April 2020 (Koh et al. 2020). These restrictions had profound impacts on the world economy (Mandel and Veetil 2020), with many workers losing their employment or wages; of those remaining in employment, many had to work from home. Recreational angling during restricted periods was often either not permitted or public access to fisheries was initially highly restricted (Midway et al. 2021). In countries and jurisdictions where recreational angling was allowed to continue in some form (but with some restrictions, such as social distancing; Paradis et al. 2021), initial angler surveys and licence sales often suggested increased participation rates (e.g. Guerra-Marrero et al. 2021; Midway et al. 2021), including first time anglers and anglers resuming after periods of inactivity (Howarth et al. 2021), and was likely related to recreational angling being considered a COVID-19 safe activity (‘social fishtancing’; Midway et al. 2021).

In addition to increased participation rates during COVID-19, there is some evidence of important changes in angler demographics and behaviours. Danish anglers who were active in restriction periods were more likely to be younger, less experienced, and more urban than prior to the pandemic (Gundelund and Skov 2021). While the number of fishing trips apparently did not increase from previous years, patterns in fishing effort shifted from weekend to weekday trips and, although catch rates were lower, more fish were retained (Gundelund and Skov 2021). Conversely, during restriction periods in Western Australia, a lower proportion of urban (metropolitan) boat anglers were active compared to regional anglers and, of those who were active, their effort was lower than before the pandemic (Ryan et al. 2021). In a global assessment of the impact of COVID-19 on marine recreational fisheries, Pita et al. (2021) reported an overall decline in activity, with negative impacts on the blue economy as well as fisher health and well-being due to the loss of fishing opportunities. Thus, whilst there were pandemic-driven changes in recreational angling participation rates, activity, and angler behaviours, these changes differed between countries and regions, and across recreational fishing methods. However, there are also substantial knowledge gaps in how recreational anglers responded to these changes in personal freedoms at global scales, despite this information being of potentially high value to fishery managers and policy makers for optimizing management and developing strategies for confronting this and other similar crises (Howarth et al. 2021).

This study aims to synthesise the temporal changes in recreational angling at global scales that relate to COVID-19 restrictions in terms of: (i) patterns of interest in recreational angling,﻿ (ii) angling licence sales, and (iii) angler participation and effort. We use these syntheses to consider how these patterns can inform the development of more resilient and sustainable recreational fisheries, particularly during future periods of global shocks (e.g. pandemics, wars). To facilitate comparisons of data, the following terms are used to define the time periods in the study and applied where possible: ‘pre-pandemic period’ (up to and including 2019),﻿ ‘acute pandemic period’ (2020), ﻿and ‘COVID-acclimated period’ (2021).

## Methods

### Interest in recreational angling: angling culturomics

We used a culturomics approach to identify temporal and spatial patterns in the global interest in recreational angling. Culturomics represents the study of human culture through the quantitative analysis of large bodies of digital data (Michel et al. 2011). It has been applied to study contemporary conservation issues through the perspective of human-nature interactions (Ladle et al. 2016; Jarić et al. 2020a, 2021). Along with ‘internet ecology’ (otherwise referred to as ‘iEcology’; Jarić et al. 2020b), culturomics has already been used to measure the various activities of anglers (Wilde and Pope 2013; Giovos et al. 2018; Monkman et al. 2018a,b; Sbragaglia et al. 2021) and to deduce whether fisheries are sustainable (e.g. McClenachan 2009; Jiménez‐Alvarado et al. 2019). Here, we used culturomic analyses to assess global changes in the volume of internet search terms related to angling activity, and then evaluated these changes regionally (USA, United Kingdom, and Australia; see Supplementary Information, Methods: Section SM1 for more detailed information on methods). We then analysed changes in the volume of internet search terms relating to angler target species, where species-specific searches were restricted to those regions in which that species was present and targeted by anglers. Data were mined for the ‘pre-pandemic period’ (2017–2019), ‘acute pandemic period’ (2020), and ‘COVID-acclimated period’ (2021; Section SM1).

### Angling licence sale data

As regulation of recreational angling often includes some form of licensing or permit system that requires prior purchase, usually from a regulatory authority (Potts et al. 2020), data on licence sale numbers and their timing were used to measure the extensive margin of angling participation. We assumed licence purchase indicated at least an intent to undertake an angling event in the near future. It was evident that (i) licence systems were not consistent between countries (e.g. variation in licence type: contrasting availability of annual, weekly and daily licences; differences in the accessibility of licence data); and (ii) there were considerable spatial differences over whether recreational angling was possible during lockdo﻿wn periods and in the immediate aftermath of their lifting. Thus, the systematic mapping of licence sales to COVID-19 restriction periods could not be attempted. Instead, licence sale data are presented for different countries grouped by continent. In all cases, licence sale data were collated from the controlling regulatory authorities of each country that is used, with these data only available from a limited number of regulators. Correspondingly, licence sale data from a specific country might not necessarily represent the licence sale trends more widely for that continent or region.

### Angling participation and effort

We used four data sources to assess changes in angling participation and effort between the ‘pre-pandemic period’, ‘acute pandemic period’ and ‘COVID-acclimated period’. The first data source was Fishbrain (www.fishbrain.com), a commercial smart-phone application (hereafter referred to as ‘app’) with > 14 M global users in 2022 who digitally log the details of their fishing events and catches. This generates fine-scale spatiotemporal data over large areas, so providing a de facto source of angling data to inform fisheries management (Venturelli et al. 2017; Cooke et al. 2021; Skov et al. 2021). A machine-learning algorithm was applied to weekly catch time and location data from Europe, North America, and Oceania (Australasia, Melanesia, Micronesia, and Polynesia) between 1 January 2015 and 1 November 2021 (see Supplementary Information, Methods: Section SM2 for specific details on the methodology used). The extracted data enabled the following metrics to be calculated for the three pandemic periods: total catches logged per week (proxy for relative fishing effort), the number of unique users who logged at least one fish per week (proxy for relative individual effort), the number of new users per week (proxy for the relative number of individuals taking up fishing for the first time), and the number of users per week who logged a catch after not logging one for at least one year (proxy for the relative number of individuals returning to fishing).

The second data source was based on data from sea angling in the United Kingdom, where approximately 2% of adults participate in this fishery (Armstrong et al. 2013). With no sea angling licence requirement, a sea angling diary scheme was implemented in 2016 to report catch data, which captures data on angler participation, effort, and catch (Hyder et al. 2020, 2021). To date, the scheme has provided catch data from over 5000 anglers and 21,600 h of angling activity since 2016 (Hyder et al. 2020, 2021). These data were used to extract relevant metrics on angling participation and effort in the three pandemic periods.

The third data source was based in Germany, where a nation-wide, representative computer-assisted telephone interview (CATI) screening survey was conducted between October 2020 and April 2021. This survey was designed to determine changes in angling activities and effort during COVID-related restrictions (Supplementary Materials: Methods, Section SM3, for detailed methodology). The survey collated general data on the proportions, socio-demographics, and heterogeneity of anglers in the German population, providing insights into changes in angling effort during restricted periods, including whether effort differed between inland and marine recreational fisheries and the effect of age, avidity, and angling skills (Section SM3).﻿

The final data source was based in Denmark, where the angling citizen science platform ‘Fangstjournalen’ was used **(**Gundelund and Skov 2021). Extracting data from this platform on angler traits of age, angling experience, and importance of angling as a hobby enabled patterns to be compared between the three pandemic periods.

## Results

### Interest in recreational angling: angling culturomics

**Fig. 1:**
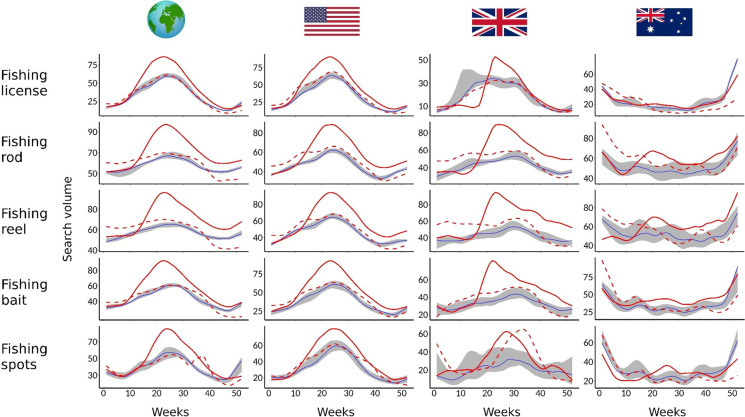
Time series of relative search volumes for the terms “fishing licence”, “fishing rod”, “fishing reel”, “fishing bait” and “fishing spots”, based on (from left to right) global Google Trends data, as well as data for the United States, United Kingdom and Australia. Full and dashed red lines represent weekly values for 2020 and 2021 respectively, while blue lines and grey shading represent median values and the range of values for the years 2017–2019 respectively. Data were fitted with LOESS smoothing (*f* = 0.2). Please note the different scales of the *y*-axes

**Fig. 2:**
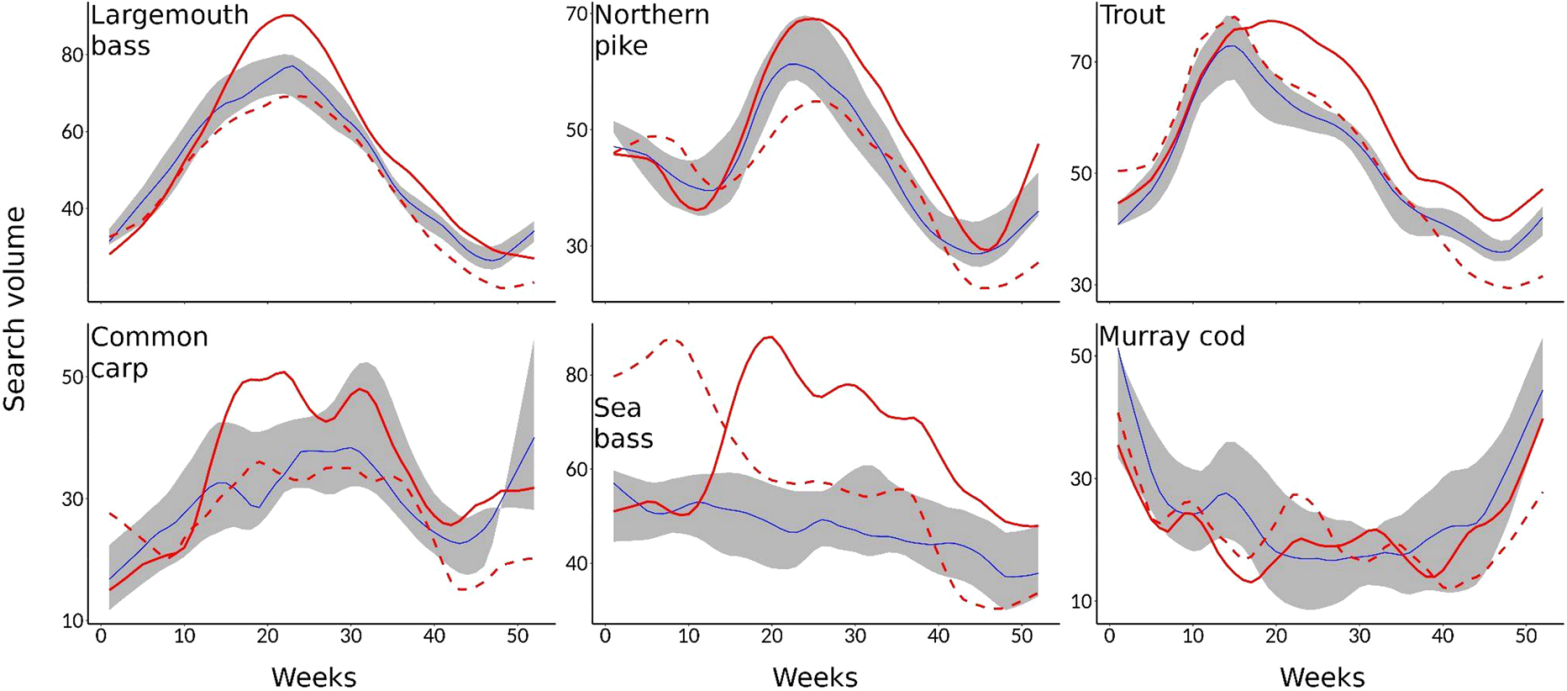
Time series of relative search volumes for largemouth bass, northern pike and trout in the United States, common carp and sea bass in the United Kingdom, and Murray cod in Australia, based on Google Trends data. Full and dashed red lines represent weekly values for 2020 and 2021 respectively, while blue lines and grey shading represent median values and the range of values for the years 2017–2019 respectively. Data were fitted with LOESS smoothing (*f* = 0.2). Please note the different scales of the *y*-axes

Overall, compared to the pre-pandemic period, there were considerable increases in global internet search volumes for all of the terms that were used as indicators of angling activity during the acute pandemic period (2020), before they returned to pre-pandemic levels in the COVID-acclimated period of 2021 (Fig. 1). This pattern was also evident in the USA and UK, where there was a clear increase in mid-2020 that generally coincided with the onset of the northern summer and the end of the initial periods of restricted periods in both countries. However, this pattern was less evident in Australia, with fewer changes in the internet search volumes in 2020, potentially due to the “Black Summer” bushfires in eastern Australia. Where there were increases, these were later in 2020, likely relating to seasonal differences between the northern and southern hemispheres (Fig. 1). There were similar increases in the volume of internet search terms for 11 of the 12 angler target species analysed, with the only exception being the Australian Murray cod, *Maccullochella peelii,* because fishing was not permitted for up to 6 months from July to December 2020 (Fig. 2, Fig. S1). With most species being focused in the northern hemisphere, peaks in searches again occurred towards the middle of 2020 (i.e. summer), but were rarely sustained in 2021.

### Angling licence sale data

Some, but not all, countries revealed substantially increased licence sales in 2020 versus other years (Table 1). In the six countries reporting increases in 2020, these were only sustained in two countries in 2021 (Table 1). Declines in licence sales tended to relate to the loss of domestic and/or international tourism.

**Table 1:** Overview of patterns in recreational fishing licence sales before and during the COVID-19 pandemic, where ‘Increase in 2020’ relates to where sales figures indicate increased licence sales in 2020 compared to previous years, ‘Sustained increase in 2021’ refers to whether any increased licence sales in 2020 were sustained, and n/a = not applicable

Continent	Country	Region/ State	Licence type	Licence duration	Years available	Increase in 2020	Sustained increase in 2021
Europe	Denmark	n/a	Mandatory freshwater/marine	Annual 7 day	2018–2021 2018–2021	Yes No (decrease during spring lockdown)	No
	England	n/a	Freshwater	All	2017–2021	Yes	No
	Germany	Saxony	Freshwater	Annual	1993–2021	Yes	Yes (but positive trend already prior to 2020)
	Germany	Bavaria	Freshwater	Annual	2001–2020	No	No
	Germany	Brandenburg	Freshwater	Annual	1990﻿–2020	Yes	Yes (but positive trend already prior to 2020)
	Germany	Mecklenburg-Western Pomerania	Coast	Annual	1990–2021	No	No
	Norway	n/a	River	Annual	2005–2020	No	n/a
Asia	Japan	Nationwide	Freshwater	All	2020–2021	No	n/a
Oceania	Australia	New South Wales	General	3 day, 28 day, 1 year, 3 year	2010–2021 (annual)	Yes	n/a
		Northern Territory	No licence	–	–	–	–
		Queensland	No licence	–	–	–	–
		South Australia	Impoundments only				
		Tasmania	Fishery/species	Annual	1995–2021 (annual)	No	n/a
		Victoria	General	48-h, 3 day, 28 day, 1 year, 3 year	2010–2021 (annual)	No	n/a
		Western Australia	Fishery/species	Annual	2010–2021 (monthly)	Yes	No
North America	Canada	Ontario	All	Annual	2012–2020	No^a^	n/a
South America	Brazil	Nationwide	All	Annual	2010–2021﻿ (annual and monthly, with interruption)	No	n/a
Africa	South Africa		Marine	Annual	2010–2020	No	n/a

^a^Note that for Ontario, resident fishing licence sales increased, but non-resident fishing licence sales decreased due to travel restrictions, hence the overall decrease

### Europe

In Denmark, annual recreational fishing licence sales by month since 2018 revealed some marked increases in the two months following a lockdown in March 2020, but this was not sustained in the longer-term, with 2021 sales generally being back to pre-pandemic levels (Fig. S2). Weekly licence sales in the same period showed an opposite effect, with sales crashing following the 2020 lockdown (Fig. S2a), presumably because these mandatory licences are mainly purchased by non-Danish tourists who were unable to travel and/or legally enter Denmark. In England, where a licence is only needed to fish in inland waters with rod and line, COVID-19 restrictions in 2020 resulted in no angling in April, partial restrictions from May to July and September to December, and no restrictions in August. There were also no restrictions in 2021. Licence sales across all types increased by 18% in 2020 compared to 2019, especially from May to July, but returned to 2019 levels in 2021 (Fig. S3). Licence sale data from Germany showed inconsistent patterns among federal states (Supplementary materials, Results: Section SR1), with sales varying little in Bavaria with no COVID-19 alteration in 2020 (Section SR1), whereas memberships of the state angler association of Brandenburg increased by approximately﻿ 7% in 2020, although memberships were already increasing prior to the COVID period (Section SR1). Coastal fishing licences in Mecklenburg-Western Pomerania, which strongly depend on domestic tourists (Arlinghaus et al. 2021), showed clear evidence of declining sales in 2020 that continued in 2021. However, licence sales in most German federal states require one-time completion and assessment of a 30-h course (von Lukowicz 1998), so short-term changes in licence sales are unlikely. In Norway, where licences are mandatory for riverine fishing for anadromous salmonids, no changes in licence sales in 2020 were apparent relative to the long-term pattern of sales (Fig. S4).

### The Americas

Canadian licence data were available for the province of Ontario (bordering USA and the Great Lakes), with sale data categorised by angler origin (Table S1). The main pattern detected in these data was a pronounced reduction in sales to non-resident (i.e. international) anglers between 2019 and 2020 (-89%), whereas licence sales to Ontario anglers increased by 10% in the same period, suggesting increased activity by anglers who had no option but to fish locally (Table S1). In Brazil, there is a requirement to purchase an annual national fishing licence for both marine and freshwater angling, with state licences also often being required (Freire et al. 2012). Due to management issues in Brazilian recreational fisheries (Freire et al. 2021), licence sale data were limited to 2010–2014 and then June 2020﻿–July 2021. These data suggested a relative decrease in licence sales during the 2020 pandemic period, followed by increases in July 2021 (Fig. S5).

### Africa

Levels of recreational angling participation in South Africa are relatively high versus other emerging economy nations (Potts et al. 2021) and, although participation and effort are not measured directly, marine recreational anglers must purchase an annual licence. Licence sale data by month suggested that sales increased by 30% between May 2020 and April 2021 versus these periods between 2015 and 2019, with peak sales in December 2020 and January 2021 (Fig. S6). While these increases were considered as being related to the COVID-19 pandemic, they might not necessarily reflect increased angling activity. For example, while recreational angling was allowed from beaches from June 2020, other activities (e.g., surfing, hiking) were not allowed until September 2020 and so some people circumvented this prohibition on non-angling activities by purchasing angling licences and carrying fishing rods on the beach (but not then fishing). Licence sales in July 2020 were low relative to 2015–2019 due to cancelled school holidays (Fig. S6). Non-angling beach activities were then banned again in December 2020 and January 2021 (summer vacation period) and when the data from these months were omitted, the average sales between 2020 and 2021 were only 2% higher than between 2015 and 2019.

### Australia

Licence requirements vary between Australian states and territories, with some not requiring licence purchase and others offering licences of up to three years duration, which makes general patterns more difficult to decipher (Table 1). Licence sales (including renewals and new licences) showed evidence of changes relating to the COVID-19 pandemic with up to an 11% increase from 2019/20 to 2020/21, for example 429,177–474,517 (+ 11%) in New South Wales, 219,896–241,773 (+ 10%) in Western Australia (WA) and 226,030–250,760 (+ 11%) in Victoria. Monthly licence sales in WA suggested increased fishing activity following the commencement of the COVID-19 pandemic. Although there were no extended lockdowns, an extended border closure prevented visitors from entering the state, with residents encouraged to “holiday at home”. Anecdotally, holiday accommodation and sales of boats and fishing equipment were in high demand. Licence sales (Recreational Boat Fishing and Rock Lobster) were above the 10-year median for most months from June 2020 and in 2021 (Fig. S7).

### Asia

A COVID-19 state of emergency was initially declared across 23 inland fisheries cooperatives around Tokyo and Osaka (Japan) in April and May 2020. The median ratio of sales of recreational fishing permits relative to April and May 2019 was only 78%, which was lower than the median of 93% for the 196 fisheries cooperatives in other regions of the country. This result indicates reduced sales, likely because restrictions of the movement of people prevented most, if not all, angling activities. However, the 2020 Japanese domestic shipment value of recreational fishing equipment, in particular marine shore fishing equipment, was the highest recorded since 2010. A small number of the inland fisheries cooperatives either postponed their opening dates of the ayu *Plecoglossus altivelis* 2020 fishing season, or did not open at all. This resulted in the ayu fishing season starting on 1 July 2020, the period just following the ending of the declaration of the state of emergency that had been in place due to COVID-19.

### Angling participation and effort

#### Spatiotemporal changes in effort inferred from the smartphone app ‘Fishbrain’

All metrics derived from the smartphone app ‘Fishbrain’ in Europe, North America, and Oceania were trending upward from 2015 to 2019 (e.g., Fig. 3, Fig. S8), which parallels an increase in the popularity of the app. Individual effort, overall effort, and both new and returning anglers were muted or declined in Oceania and, to a lesser extent, Europe during both the acute and COVID-19 acclimation periods (Fig﻿. 3, Fig. S8). These trends are consistent with bans or limits on recreational fishing and/or both domestic and intercontinental travel. Indeed, Australia’s border closure prevented recreational fishing by international tourists, which represented 6% of the reported Fishbrain catches from this region in 2019. In contrast to Oceania and Europe, individual effort, overall effort, and both new and returning anglers all increased in North America (Fig. 3, Fig. S8). Increases were strongest in 2020, and either weakened or disappeared in 2021.

**﻿Fig. 3:**
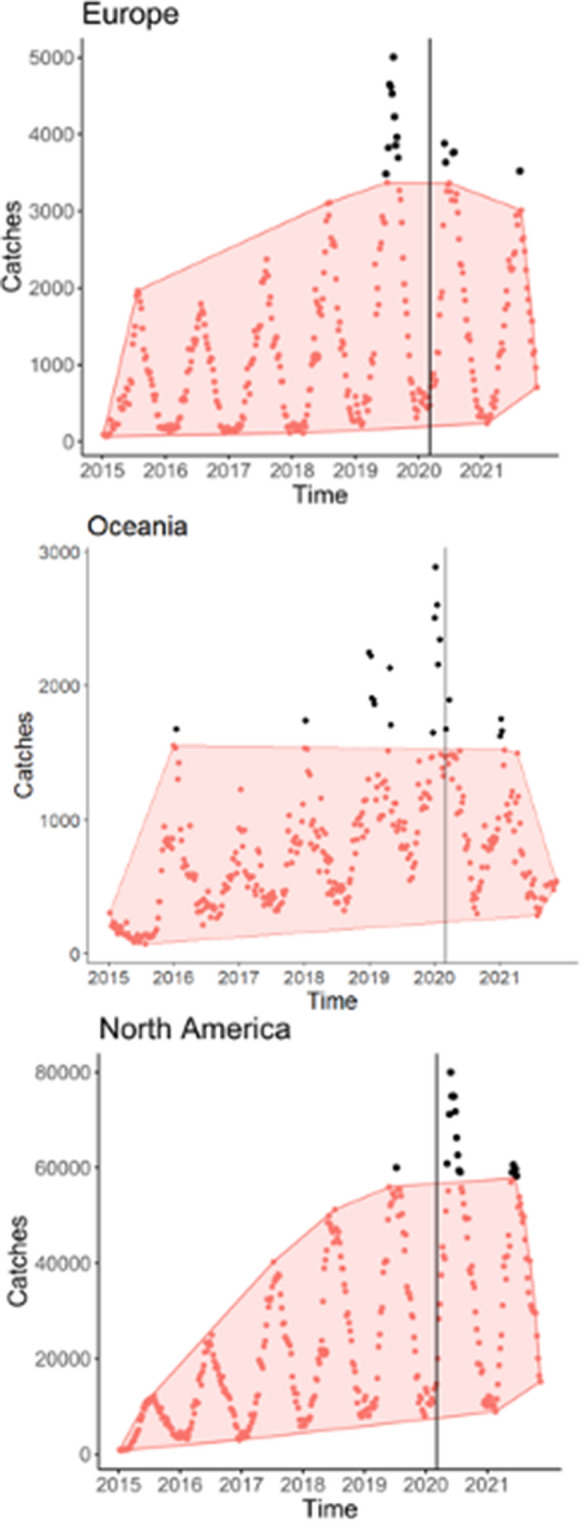
Weekly catches of all marine and freshwater species by continent reported via the Fishbrain smartphone app from 1 January 2015 to 31 October 2021. The shaded area in each panel identifies the collective pattern of the majority of data points, black points are anomailies that do not follow this pattern (see Section SM2 for details), and the vertical black lines signify when the acute pandemic period commenced

### Changes in UK sea angling effort

Sea angling effort was reduced in the lockdown period of spring 2020 versus 2019 in the UK, which then increased to above 2019 levels in July to September 2020 as restrictions relaxed, and then returned to similar levels from October 2020 (Hook et al. 2022). The anglers also travelled less far to fish in 2020 than 2019 (Hook et al. 2022). A sub-set of these anglers were also surveyed and revealed reductions in their expenditures, physical activity, and well-being related to sea angling during the acute pandemic period (Hook et al. 2022).

### Changes in angling effort in Germany

**Fig. 4:**
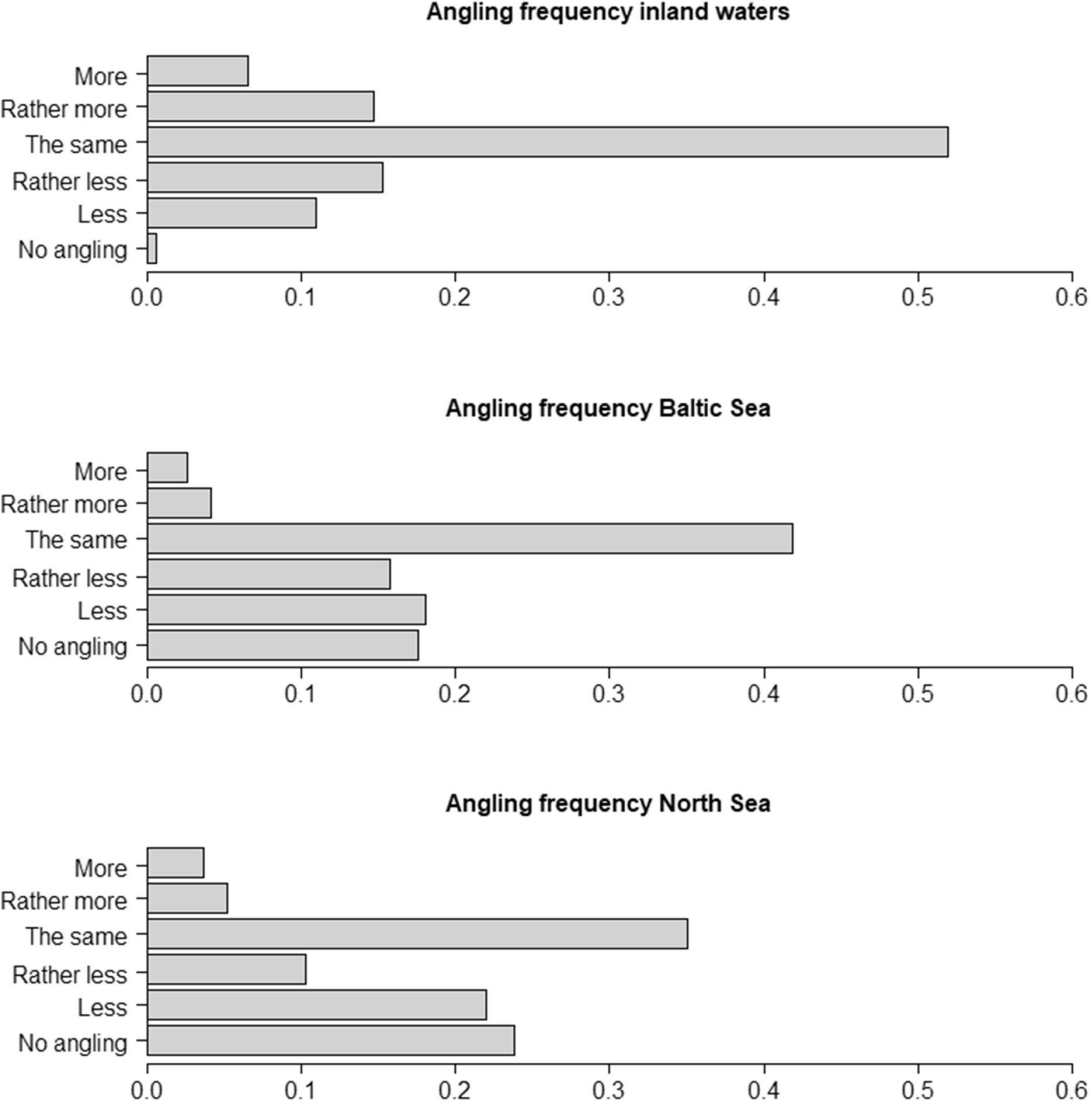
Angling frequencies in German inland and marine (Baltic Sea and North Sea) waters during the COVID-19 restriction period from March to May 2020 as stated by participants of the telephone survey (weighted data)

Of the 2,792 anglers interviewed in the German survey (Supplementary Materials Results: SR2), ~ 60% had fished during the acute pandemic period, with most inland anglers fishing as frequently as in the pre-pandemic period. Only 21% of the inland anglers fished more than usual. Although inland anglers generally continued their fishing activities, > 50% of those fishing in marine waters of the Baltic and North Sea reduced or stopped fishing during the acute pandemic period (Fig. 4), most likely due to travel restrictions and many of these anglers being domestic tourists (Strehlow et al. 2012; Lewin et al. 2021). This result agreed with the reduced coastal fishing licences sold for the state of Mecklenburg-Western Pomerania (Section SR2), suggesting that German tourism-based marine recreational fisheries suffered strongly from COVID-19 related declines. Comparisons of anglers in the urban federal state Berlin and anglers from the surrounding, more rural federal state Brandenburg revealed that most anglers in both states fished as usual during restrictions in the acute period, with no significant differences versus the pre-pandemic period (Section SR2). However, angler demographics shifted towards younger and more avid/specialised anglers in restricted periods (Section SR2). Anglers were also more likely to continue fishing in restricted periods if they were members of angling clubs, and these continuing anglers were also more likely to consider their angling skills as above average.

### Angling citizen science platform in Denmark

Comparisons of Danish angler traits (age, angling experience, and importance of angling as a hobby) in periods of pre-pandemic (2018, 2019), acute pandemic (2020) and COVID-acclimated (2021) revealed that angling participants in 2020 were younger, less experienced, and more urban-based than participants in 2018 and 2019, but with anglers in spring 2021 being more similar to pre-pandemic participants (Figs. S9, S10). Thus, the 2020 shifts in angler characteristics represented a short-term response to the COVID-19 restrictions.

## Discussion

There were some strong pandemic-related changes in the behaviour of individual anglers and overall patterns of interest, effort, and engagement, with trends including a peak in online interest in recreational angling in 2020, shifts in angler behaviours and demographics, and some changes in licence sales. While these trends varied by region, they generally reflected country-specific COVID-19 restrictions and long-term patterns of sales and activity (e.g., loss of international anglers, loss of domestic travellers, and increased numbers of younger anglers in 2020). There is greater uncertainty as to whether local effort changed (although most angling effort was likely to be more locally based due to travel restrictions), but more evidence indicated that changes were highly transitory and quickly reversed by 2021.

There were some context dependencies in these results and evaluations at country and regional levels. In entirety, however, we argue that they provide a series of considerations that can contribute to the development of more resilient, responsible, and sustainable recreational fisheries (Cooke et al. 2019). Of course, there is high uncertainty regarding the COVID-acclimated period and what it will mean for recreational anglers and fisheries management. Nonetheless, we also suggest that it is important to develop a suite of considerations that are relevant to various actors who are involved in the recreational fishing sector including anglers, the industry (including outfitters and guides, manufacturers, and popular media), scientists, fisheries managers and regulators, and conservation bodies. We acknowledge, however, that transitions to periods of living with COVID-19 will vary extensively based on geopolitical, cultural, and economic factors with, for example, access to vaccines and health care remaining more limited in the global south. Correspondingly, some of our considerations outlined below might not be universally applicable.

*Expand licensing programmes where relevant:* Angling licence regulations vary across the world, with many regions where recreational licence programmes do not exist (Bower et al. 2020). Moreover, even where they do exist, sale data are often unavailable or unreliable. We thus suggest that jurisdictions without licensing should consider instituting (or resuming) a licence system to better understand and manage recreational fisheries in the post COVID-acclimation period, given licences (including permits or registries) provide a measure of participation and potential fishing effort. Licences also provide a sampling frame for gathering data that can inform catch, social and economic fishery objectives, which collectively support access-rights and allocation to shared fishery resources, and also provide a means for communicating regulations and services, and potential revenue to improve recreational fishing. Many regions and jurisdictions with licence programmes invest a proportion of the associated revenue into recreational fisheries management (Organ et al. 2012; Peterson and Nelson 2017; Skov et al. 2020; Sect. 3). However, compliance with licence requirements can be low (e.g. Spain: Gordoa et al. (2019); Brazil: Freire and Rocha (2021)). B﻿roader recreational fisheries governance, including licensing, might improve compliance (Arlinghaus et al. 2019; Potts et al. 2020).

*Normalise the use of culturomics to assess trends in fishing interest:* Culturomics are increasingly being used in conservation and natural resource management (Ladle et al. 2016) and are being embraced for aquatic issues (Jarić et al. 2020a,b). Normalising their use in recreational fisheries management should help to provide more timely data on regional, national and/ or global trends (Lennox et al. 2022). These tools open up a wide range of opportunities for acquiring new types of data on angling activities, providing managers with data on effort and catches that were previously unavailable (Jarić et al. 2021; Lennox et al. 2022), although there is a concomitant need to determine the accuracy and precision of the patterns they show more generally, such as whether increased search volumes translate into elevated angling effort.

*Develop strategies for angler retention and education on responsible fishing practices:* The pandemic resulted in many new and reactivated anglers in some countries, so attempts to retain these anglers are important in COVID-acclimation periods. Creating opportunities for new anglers to integrate into recreational fishing communities (e.g., links to angling clubs, organisations, and via social media) should help retention, while also serving as a vehicle for instilling responsible fishing practices and knowledge transfer related to regulations and other management tools. Indeed, anglers who were associated with angling clubs in Germany remained most active during periods of movement restrictions (see “Angling licence sale data” section).

New and reactivated anglers may be less likely to befamiliar with responsbile fisheries practices, local regulations, or consumption advisories, especially as regulations tend to be complex and open to interpretation. From basic fish identification to understanding the complexities of regulations, there is a need to ensure that new anglers understand how to comply with fisheries regulations. For example, Page and Radomski (2006) found compliance with recreational fishing regulations lowest among novice anglers in Minnesota, USA. New anglers may also be unfamiliar with best handling practices (Brownscombe et al. 2017), which are intended to minimise welfare impacts and post-release mortality. New anglers may also be unfamiliar with the complexities of using live baitfish and the risks associated with the inter-basin transport and release of baitfish or other live bait (e.g., Drake and Mandrak 2014; Lewin et al. 2019). These challenges can be overcome via education and outreach initiatives, including mandatory courses associated with licensing (Sexton 2021), workshops (e.g., Delle Palme et al. 2016), social movements (e.g., Keep Fish Wet; Danylchuk et al. 2018), and a full behavioural strategy (Mannheim et al. 2018). There are also opportunities to consider how nudges (Mackay et al. 2018) and sanctioning (Guckian et al. 2018) could result in more responsible recreational fishing practices.

*Create more urban fishing opportunities:* There was increased use of urban angling opportunities in some regions as COVID-19 movement restrictions were lifted, especially among younger anglers. Increased urbanisation (United Nations 2018) more generally also means that urban fishing is a growth opportunity in many jurisdictions (Arlinghaus and Cooke 2009; McPhee 2017). As not all individuals have access to private, motorised transportation, access to fishing close to home is an equity issue. While urban fisheries are not new, we emphasise here that they ﻿were timely given increased interest in fishing during the acute pandemic period, and other events in future that could result in similar heightened interest in urban fishing. Fishing programs in urban areas can also be used to both recruit and retain urban anglers (Balsman and Shoup 2008). The extent of urban angling opportunities available in Berlin, Germany, meant that anglers there were able to engage in recreational angling in a similar manner to those in the surrounding rural areas. Thus, considering urban fishery resources within long-term strategies to increase angling participation is important, especially as urban anglers tend to be more avid and committed than rural anglers (Arlinghaus and Mehner 2004).

*Consider how emerging recreational fishing can be used to support livelihoods:* Recreational fishing generates substantial economic benefits that support regional economies and individual livelihoods. Some COVID-19 pandemic restrictions (e.g., limits to shopping at fishing tackle stores, limits on access to some fishing sites, prohibition of guiding or competitive events, limits on cross-jurisdiction tourism) had negative impacts on the recreational fishing industry and its members, although some sectors might have boomed (e.g. online stores). As jurisdictions transition to COVID-acclimation periods, consideration is needed to ensure that relevant sectors can remain vibrant and support livelihoods while also ensuring angling participants can continue to access the services that they require. Achieving this might need policy adaptation through promotion of a “whole of government” approach, and the development of recreational fisheries-directed facilities and services (Potts et al. 2022). Moreover, benefits from recreational fishing in a given area should generate benefits for that area rather than having them accrue to interests located elsewhere (e.g., in a different community, different state or even different country; Bower et al. 2014; Barnett et al. 2016; Butler et al. 2020). Mapping this economic benefit back to data on angler engagement (e.g., licence and permit sales) is important to understand the relationship between angling economics and angler engagement.

*Create more opportunities for angler citizen science and co-management:* App-based effort and harvest data became the sole data source in many regions during the pandemic, reflecting the growing number of ways anglers can engage in fisheries assessment and management that were also apparent pre-pandemic. For example, various citizen science programs such as angler diaries (Cooke et al. 2000; Hyder et al. 2020; 2021) and phone-based angler apps (Venturelli et al. 2017) were already providing opportunities for anglers to share their observations with fisheries managers (e.g., on stock assessment and angler behaviour), and then provided data during the pandemic (e.g., Gundelund et al. 2021b, 2022; Skov et al. 2021). There is a general need for and great potential in citizen science programmes that also invite and involve recreational fishers in co-management initiatives (Arlinghaus et al. 2019). Expanding such efforts in the case of future pandemics and to support modern fisheries management is timely. The use of remote methods to capture information and data was also timely given the pandemic impacted efforts to engage anglers in fisheries management activities as face-to-face meetings were paused. Reinstating such efforts rapidly to create pathways for angler engagement and consultation in fisheries management processes will be important to ensure diverse perspectives are considered (e.g. those unwilling or unable to engage with App-based technologies) (Elmer et al. 2017), plus these efforts build support and understanding for fisheries management actions (Reed 2008; Dedual et al. 2013). When these processes are integrated with apps and citizen science platforms, there are substantial opportunities for two-way communication between anglers and managers (Venturelli et al. 2017).

## Conclusions

The global COVID-19 pandemic altered many aspects of human society, especially in relation to freedoms and economic activity (from local to global levels). We have demonstrated here that some considerable changes in angling interest, behaviour and effort also occurred in this period. While acknowledging that the licence and effort data were imperfect, we argue these data emphasise the importance of overcoming these imperfections through improved data collection in recreational fisheries more generally. Indeed, the acknowledgement that there remain substantial gaps in our understanding of how recreational anglers respond to changes in social circumstances more generally is a major step forward in understanding how to overcome these issues. The collated information did demonstrate that, whilst there was some high spatial variability in some results, there were some distinct patterns in both licence sales and effort, including the loss of tourist anglers. Furthermore, people also had to fish more locally, with these people often being younger than most anglers who fished prior to the pandemic. In some regions, however, we have also seen a rapid transition back to pre-pandemic conditions, suggesting that the changes were not sustained. Thus, despite the apparent attractiveness of recreational angling during periods of high individual and societal stress, the retention of new anglers remains elusive. Managers and policymakers can use the data and considerations here to ensure that their fisheries remain accessible for the benefit of anglers of all ages and abilities, while ensuring that these fisheries can be used to help recruit and retain new anglers—irrespective of the overriding economic and public health conditions.

## Supplementary Information

Below is the link to the electronic supplementary material.

Supplementary file1 (DOCX 1126 kb)

## Data Availability

Data are available from the authors on reasonable request.
