# Supplementary material for: Global responses to the COVID-19 pandemic by recreational anglers: considerations for developing more resilient and sustainable fisheries
Source: Rev Fish Biol Fish. 2023 May 30:1–17. Online ahead of print. doi: 10.1007/s11160-023-09784-5 (PMC10227408; doi:10.1007/s11160-023-09784-5)
Supplement: Supplementary file 1 — Supplementary file1 (DOCX 1126 kb) [file 11160_2023_9784_MOESM1_ESM.docx]

**Supplementary materials - Britton et al. Covid 19 & recreational angling**

This file comprises of:

Supplementary material on methods (SM)

Supplementary material on results (SR)

Supplementary Tables (Table S.x)

Supplementary figures (Fig. Sx)

**Supplementary material on Methods (SM)**

**Section SM1: Angling culturomics**

We explored public interest in recreational fishing during the COVID-19 pandemic (2020-2021) based on relative search volumes from the Google search engine. Data on weekly relative search volumes were downloaded from the Google Trends platform (https://trends.google.com) for selected search topics, which included general fishing terms that are expected to reflect public interest in recreational fishing (i.e., fishing licence, fishing rod, fishing reel, fishing bait, and fishing spots, *cf*. Figure 1), as well as names of popular angler target species (Figures 2 and S2). For fishing terms, we assessed global search volumes, as well as data restricted to selected countries (United States, United Kingdom, and Australia), while search volumes for fish species were assessed for selected countries within their respective ranges. All searches were made using Google Trends "topics", which provide combined results for all related terms, including synonyms, multiple names (i.e., both vernacular and scientific species names), different spelling versions, misspellings, as well as results across languages. The only exception was the term "fishing spots", which was not available as a topic, and the search was based on a regular search term that provides results only for the specific term searched. Trends in public interest were compared with the period prior to the pandemic (2017-2019), as a control group.

**SM2: Spatiotemporal changes in effort inferred from the smartphone app ‘Fishbrain’**

We used ArcGIS Pro version 2.8.2 to assign the geographic coordinates of each Fishbrain catch from 1 January 2015 to 31 October 2021 to one of the seven continents if it was within 50 km of that continent. Catches that were not within this buffer region were ignored, and catches that were associated with multiple continents were assigned to the continent that they were closest to. We used the time stamp that was associated with each catch to assign it to a week of the year (1-52) starting from 1 January. The last week of each year comprised 8 days from 24-31 December (or 9 days from 23-31 December in a leap year).

We excluded Africa, Antarctica, and Asia from our analyses because annual catch counts in these continents were relatively low (just hundreds to thousands vs tens of thousands to over a million in other continents) and therefore less likely to be representative. We also excluded South America because of the potentially confounding effects of a steady decline in app use for 2-3 years prior to the pandemic.

We used Density Based Spatial Clustering of Applications with Noise (DBSCAN) to identify continent-specific changes in each of our five metrics. DBSCAN is a clustering-based, unsupervised machine learning approach that is appropriate for time series trend and anomaly detection (Ester et al. 1996). We applied DBSCAN separately to each combination of continent and metric via the DBSCAN package (Hahsler et al. 2019) in version 4.1.1 of the R statistical package (R Core Team 2021). The algorithm requires users to specify epsilon (the radius of the neighborhood around a point), and the minimum number of neighbors that are within the epsilon radius. We set the minimum to 10, and then used a k-nearest neighbor distances approach to estimate the optimal epsilon value. This approach involved calculating the average of the distances of every point to its k nearest neighbors, plotting the resulting k-distance curve, and then identifying the epsilon value that was associated with the steepest bend in the curve. We also specified that the data were unscaled and raw when conducting each DBSCAN analysis. Finally, we re-ran all analyses to confirm that results were robust to an order of magnitude in either direction of minimum = 10.

**SM3: Changes in angling effort in Germany – Materials and Methods**

The survey used a dual frame approach with 70% landline numbers and 30% mobile numbers. A mixture of random-digit dialing (RDD) and number sampling from an official number registry (landline numbers only) was used to derive telephone numbers and contact households, with selection probabilities being proportional to the number of households per municipality. However, a disproportional sampling approach was chosen to increase the number of marine anglers for a subsequent diary study. Therefore, the probability of sampling telephone numbers originating from 8 out of 16 federal states that are closer to the German coasts was doubled.

A total of 1,541,182 telephone numbers were used to realize 150,232 interviews. Of these numbers, 683,135 (~44%) were mobile numbers and 858,047 (~56%) were landline numbers. Up to ten attempts were made to contact an individual household. Thereafter, a telephone number was considered a quality-neutral failure. An angler was defined as a person who had fished at least once in Germany during the last 12 months preceding the survey. Survey participants had to be older than 14 years due to the German Youth Protection Act. All identified anglers that had been fishing in Germany in the last 12 months, or who planned to go fishing there in the next 12 months were asked to participate in a 15-minute interview. Apart from questions regarding angling effort (number of fishing days in the past 12 months, “avidity”), socio-demography (e.g. age, sex, residence), and angler heterogeneity (skill level, centrality, catch orientation), three questions regarding potential effects of the COVID-19 pandemic on individual angling effort were asked. The first two questions with binary responses aimed to evaluate if the participants went fishing during the period of the first lockdown in Germany (March-May 2020) and if not if this was due to the restrictions. The specific questions were: “Did you go fishing during the period of the COVID-19 restrictions in March, April and May 2020?” and “Did you not fish during the period of the COVID-19 restrictions (March, April, May 2020) due to the restrictions or because you would have not gone fishing anyway?”. The third question asked the participants to rate the development of their angling effort for marine and inland waters during the COVID-19 restrictions based on a five-point Likert scale ranging from: (1) less fishing to (5) more fishing. This question was not presented to participants that did not go fishing at all in the last 12 months preceding the CATI interview or who had indicated that they did not go fishing during the restriction period because of other reasons. The specific question was: “Did you go fishing (1) less/ (2) rather less/ (3) the same/ (4) rather more/ (5) more during the period of the COVID-19 restrictions in March, April and May 2020?”.

For the analyses, only data from anglers who went fishing in the 12 months preceding the CATI survey as well as anglers who did not go fishing because of the COVID-19 restrictions were included. Data from anglers who did not provide complete data or did not go fishing because of other reasons were not considered. Prior to the analyses the data were weighted using individual weights for each angler. These weights accounted for the disproportional sampling approach using a multiplier. Furthermore, the distribution of household sizes in Germany from the German micro census 2018 (<https://www-genesis.destatis.de/genesis/online>) was used to adjust the distribution of household sizes found in the CATI survey data to minimise design-based biases.

The analyses were conducted using the R statistical language version 4.0.2 (R Core Team, 2020 and the R package “survey” (Lumley, 2020).

**Supplementary materials on Results (SR)**

**SR1. Licence sales in Germany**

There is no access to national-level information on COVID-related changes in either angling licences or general angling effort. Moreover, analysis of angling licence development does not necessarily mean that angling effort has changed, as individual people that are already licence holders may simply change their effort during the COVID-19 pandemic.

The picture about angling licence holders varied by federal state in Germany. Analyses of a few sample cases does not indicate a uniform pattern and calls into question the presence of substantial and sustained nationwide changes. Specifically, it is unclear whether there is a consistent COVID signal in some of the data as the developments during the pandemic mirrored trends already visible prior to the pandemic. For example, in Bavaria licence numbers have been quite stable since the early 2000s, with no evidence for COVID-related rises in 2020. By contrast, membership numbers of anglers in the state association of Saxony have been continuously rising since the early 2000s, such that no peak in the fishing season 2020 can be differentiated from the general underlying trend of increased angler interest (Fig. SR1). The coastal fishing licence of Mecklenburg–Western Pomerania showed a decline in 2020 that was further aggravated in 2021, but declines were seen already since 2015 and thus the trend has been negative before the pandemic started. In Berlin, analysis of youth angler licences indicated a steep decline over the past decade with no change in response to COVID (Arlinghaus, unpublished data). As a final and clearer example, the state angler association of Brandenburg has reported a rise by 7,000 members in the first COVID-19 year 2020 (which represents a rise of about 7%), which they think is a direct response to the increased attractiveness of recreational fishing as typically only rises among 1000 and 2000 people are registered (https://www.berlin.de/aktuelles/brandenburg/6417009-5173360-coronatrend-angeln-boomt-in-brandenburg.html). However, as in the case of Saxony, the positive trend was already initiated before 2020 and may only be reinforced by the pandemic. Note that in most German states there is a legal demand to pass an angling examination before being able to get an angling licence. The courses were often on hold in response to the COVID pandemic in 2020, preventing people from quickly acquiring new licences. States relying on substantial domestic tourists, such as Mecklenburg–Western Pomerania, likely lost tourists, which could in part explain the declines in licences in 2020 and 2021. At the same time, however, changes to daily bag limits for cod *Gadus morhua* fishing and declines in local pike *Esox lucius* stocks at the Baltic coasts (van Gemert et al. 2022) already reduced the attractiveness of Mecklenburg-Western Pomerania as a destination, contributing to the declines we see in the licence sales. Overall, the impact of the pandemic on the licence sales in Germany is far from being obvious and unlikely to be positive in the long-term.


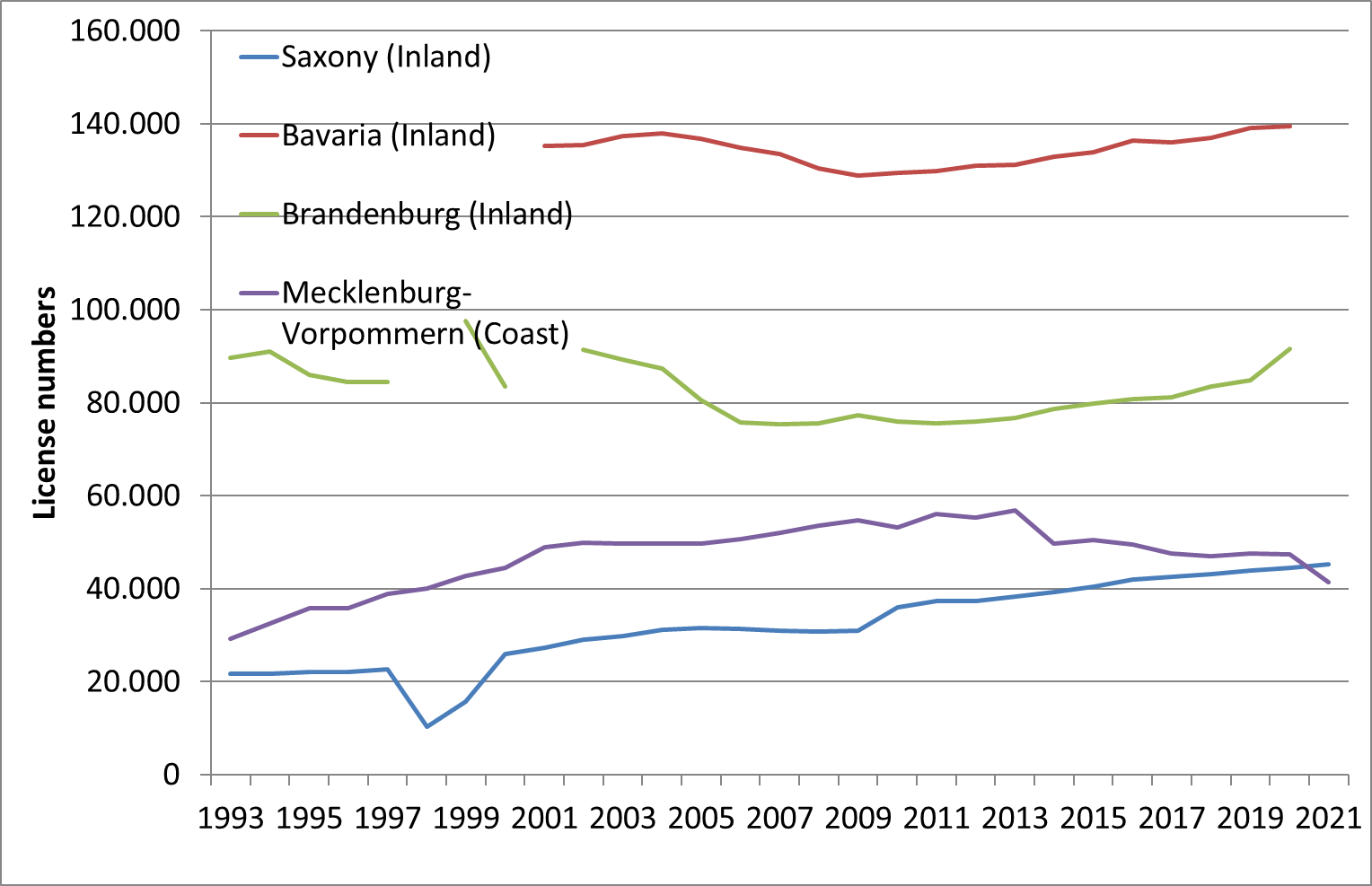


Figure SR1. Development of licence sales in four German federal states. The data for Brandenburg, Bavaria and Saxony are for inland states and the data for Mecklenburg–Western Pomerania are annual coastal fishing licences for adults. Completed data for 2021 only exist for the coastal licences, while the figure in Saxony reflects the status at the beginning of 2021 only.

COVID might have also affected the available time of those that were already licence holders and thereby affect effort. Again, as in the case of the licences, different trends have likely happened in areas with a lot of local angling potential compared to areas that depend on angling tourism from other states in Germany. For example, in the area around Berlin, there was a subjective rise in local angling effort as many lakes and river sections were intensively used during the lockdown, but no quantitative estimates are available. Surveys mentioned above suggest that it was mainly the more avid people that increased effort, while the majority of surveyed anglers in Berlin and Brandenburg suggested similar fishing effort as before the pandemic. Standardized survey data collected post COVID on coastal angling for northern pike in northeastern Rügen as well as for cod angling in the Baltic Sea, however, suggest a different picture (Kömle, Bronnmann and Arlinghaus, unpublished data). In the pike fishery of the German Baltic, roughly 2,000 respondents to a survey indicated that only about 20% of licence holders fished more after the start of the pandemic, of which only 35.9% said that the reason for increased time availability in response to the pandemic. One third of anglers said they fished less often than normal at the coast for pike in response to the pandemic, of which 44.5% said that a core reason was less time availability due to the pandemic. Similarly, in relation to cod angling, 75% of about 2,000 surveyed anglers indicated they fish less or much less after the onset of the pandemic. Both cases of Baltic pike and cod angling involve substantial proportions of travel among German states (Lewin et al., 2021), which was difficult and partly prohibited for leisure reasons due to the lockdown.

Overall, during the lockdown the access to recreational fishing remained largely possible, but most club activities were not possible. Also, over multiple months travel among states was difficult and angling shops were closed so that access to gear and licences was difficult or impossible. In some states, agencies also banned recreational fishing for a short period of time when measures were taken to increase social distances. In the case of Berlin, the ban on recreational fishing was lifted after two days and after immense critique by angler associations, but the constraints on between state travel (e.g., people in Berlin traveling to, say, Bavaria to fish) still meant that the possibilities to fish outside local scales was severely reduced over multiple months. Only work-related travel was possible, which meant that many tourism-dependent fisheries were largely devoid of fishing pressure over multiple months.

**SR2. Changes in angling effort in Germany: Results from self-reports**

A total of 5,763 households with at least one angler were identified during the CATI survey and a total of 2,792 interviews with anglers were completed. The majority of the anglers (72% of the participating anglers) fished in inland waters followed by the Baltic Sea (23%) and the North Sea (5%). About 61% of the interviewed anglers were members in German angling clubs or associations.

*Changes in angling frequencies in inland and marine (Baltic Sea and North Sea) waters during the COVID-19 restrictions*

Approximately 18% of the interviewed anglers have not been angling during the 12 months preceding the CATI interview, 60% of the anglers went fishing during the COVID-19 restrictions. In contrast, about 14% stated that they had not been angling because of the restrictions whereas 25% had not been angling anyway. The angling activity during the restrictions, however, differed between inland waters, the Baltic Sea and the North Sea (*Chi*² test, *F* = 267.3, d.f. = 10, *p* < 0.0001, Fig. 3 main text).

More than 50% of the interviewed anglers continued fishing in inland waters during the restriction period, but at similar levels as before. About 21% fished more than usual, whereas about 27% fished less than before the COVID-19 restrictions came into place. Less than 1% of the inland anglers stopped fishing during the restriction period (Table SR2.1). Also, in the Baltic Sea and North Sea, a great part of the anglers went fishing as usual. In contrast to the inland recreational fishery, more than 50% of the interviewed anglers reduced or stopped fishing during the restriction period and only about 7% and 9% of the anglers fished more frequently on the Baltic Sea and on the North Sea coast, respectively (Table SR2.1). These results can most likely be attributed to the fact that many marine anglers in Germany are domestic tourists (Strehlow et al., 2012; Lewin et al., 2021) who travel from other German federal states to the coast and were therefore more affected by travel restrictions than inland anglers who may fish in their home state.

Accordingly, whereas the stated angling frequencies of Baltic Sea and North Sea anglers were similar (*Chi*² test, *F* = 3.7, d.f. = 5, *p* = 0.6) there were significant differences of the angling frequencies between inland and Baltic Sea anglers (*Chi*² test, *F* = 234, d.f. = 5, *p* < 0.0001) and between inland and North Sea anglers (*Chi*² test, *F* = 212.2, d.f. = 5, *p* < 0.0001).

*Differences between the angling activities in rural and urban regions of Germany during the COVID-19 related restriction period*

To investigate whether the angling frequencies during the COVID-19 related restrictions differed between rural and urban anglers, data on anglers from the urban federal state Berlin (BE) and anglers from the surrounding more rural federal state Brandenburg (BB) were compared. In both areas, the majority of anglers went angling as usual (Table SR2.2; Fig. SR2.1).

Few anglers from BE stopped fishing over the restriction period. About 22% and 38% of anglers from BB and BE reduced their fishing activities whereas approximately 19% and 4% increased their activity, respectively. An overall Wald test of associations, however, indicated that the angling frequencies between both angler groups (urban and rural) were not significantly different (*F* = 1.75, d.f. = 201, *p* = 0.1). The similarity of the angling frequencies of both angler groups can be most likely attributed to the fact that Berlin offers sufficient fishing opportunities because more than 20 lakes and several rivers and channels cover about 7% of the surface of the city.

*Effects of age, avidity, skill level and membership in an angling club or association on changes in angling frequencies in inland and marine waters during the COVID-19 restrictions*

In general, anglers who went fishing during the restrictions were slightly younger (design-based Kruskal-Wallis (KW) test: *t* = -3.0, d.f. = 2,075, *p* = 0.003) and also more avid (KW test: *t* = 17.6, d.f. = 2,075, *p* < 0.0001) than anglers who stopped fishing (Table SR2.3).

Compared to anglers who were not organized in angling clubs, a significantly higher proportion of organized anglers continued fishing during the restrictions (*Chi*² test, F = 56.8, d.f. = 2,076, *p* < 0.0001; Table SR2.3).

When separated by waterbody, a more differentiated picture could be obtained. Inland anglers who increased their fishing activity during the restrictions were significantly more avid than those who reduced their activities while inland anglers who stopped fishing during the restriction period had the lowest avidity (Table SR2.4).

In contrast, Baltic Sea and North Sea anglers who reduced or stopped fishing had a higher avidity than those who fished the same as usual or slightly more. Only North Sea anglers, who fished more than usual had a higher avidity. As mentioned above, these results can most likely be attributed to the fact that many highly avid marine anglers are domestic tourists who travel to the coast and were therefore more affected by travel restrictions than local residents.

A larger proportion of anglers who continued fishing during the restriction period rated their angling skills as above average compared to those who did not go fishing (design-based Wald test of association, F = 6.7, d.f. = 2,073, *p* < 0.0001, Table SR2.5; Fig. SR2.2).

Nearly 42% of all interviewed inland anglers perceived their angling skills as above average or expert and continued fishing as before or increased their fishing activity during the restrictions while approximately 14% of the anglers decreased their activity or stopped fishing although they considered their angling skills to be above average or high. In contrast, about 8% of anglers rated their skill level as novice or below average and continued or increased their fishing activity whereas 3% of anglers rated their angling skills as below average and decreased or stopped their fishing activities (Table SR2.6). However, the fact that 29% of the inland anglers who rated their skill level as below average (low or novice) and 25% of those who rated their skill level as above average (above average or expert) decreased or stopped their fishing activity during the restrictions indicated that the effect of the self-assessed angling skill on the fishing activity in inland waters was low.

Investigating Baltic Sea anglers, about 31% of them perceived their angling skills as above average or expert and continued fishing as usual or increased their fishing activity during the restrictions while approximately 36% decreased their activity or stopped fishing although they considered their angling skills to be above average or high. About 5% of the Baltic anglers rated their skill level as novice or below average and continued or increased their fishing activity whereas 2% rated their angling skills as below average and decreased or stopped their fishing activities (Table SR2.6). About 31% of Baltic Sea anglers who rated their skill level as below average and 53% of those who rated their skill level as above average decreased or stopped their fishing activity during the COVID-19 related restrictions. This observation might reflect that angling tourists consider their angling skills as above average. No individual groups were distinguished among the North Sea anglers, because the number of interviewed North Sea anglers who had rated their skill level as novice and the number of those who rated their angling skills as below average was low (*n* < 10).

Table SR2.1 Angling frequencies in German inland and marine waters (Baltic Sea and North Sea) during the COVID-19 restriction period from March to May 2020 as stated by participants of the CATI survey (weighted frequencies and percentages).

| Angling during restrictions | Inland waters | Proportion (%) | Baltic Sea | Proportion (%) | North Sea | Proportion (%) |
| --- | --- | --- | --- | --- | --- | --- |
| No angling | 7.5 | 0.6 | 53.6 | 17.6 | 13.1 | 23.8 |
| Less than usual | 141.9 | 10.9 | 55.1 | 18.1 | 12.1 | 22.0 |
| Rather less than usual | 198.8 | 15.3 | 48.1 | 15.8 | 5.7 | 10.3 |
| The same as usual | 674.1 | 51.9 | 127.6 | 41.8 | 19.3 | 35.0 |
| Rather more than usual | 190.9 | 14.7 | 12.7 | 4.2 | 2.9 | 5.2 |
| More than usual | 85.3 | 6.6 | 7.9 | 2.6 | 2.0 | 3.7 |

Table SR2.2 Angling frequencies in inland waters during the COVID-19 restriction period (March to May 2020) in the two German federal states Brandenburg (BB; rural) and Berlin (BE; urban) (angler numbers in weighted frequencies and proportions).

|  | BB |  | BE |  |
| --- | --- | --- | --- | --- |
| Angling in inland waters during the COVID-19 restrictions | Frequency | Proportion (%) | Frequency | Proportion (%) |
| No angling | 0.00 | 0.0 | 0.43 | 1.1 |
| Less than usual | 6.16 | 9.7 | 6.10 | 16.1 |
| Rather less than usual | 7.49 | 11.8 | 8.22 | 21.7 |
| T he same as usual | 37.85 | 59.4 | 19.63 | 51.9 |
| Rather more than usual | 8.06 | 12.7 | 1.88 | 5.0 |
| More than usual | 4.11 | 6.5 | 1.58 | 4.2 |

Table SR2.3 Weighted number of anglers, avidity, age (mean ± standard error (S.E.)), and membership in angling clubs/associations grouped by the angling activity during the COVID-19 restriction period (March to May 2020).

| Angling | Frequency | Avidity | Age | Member | No member |
| --- | --- | --- | --- | --- | --- |
| Angling during restriction | 1355.4  60% | 40.1  (± 1.5 S.E.) | 48.3  (± 0.5 S.E.) | 895.4  53% | 460.0  27% |
| No angling because of restrictions | 322.2  14% | 11.5  (± 1.2 S.E.) | 51.0  (± 0.9 S.E.) | 128.3  8% | 193.6  12% |

Table SR2.4 Avidity (weighted mean ± standard error) and angling frequency of German inland, Baltic Sea and North Sea anglers during the COVID-19 restriction period (March to May 2020) including a summary of the test statistics.

| Angling in inland or marine waters during the restrictions | Avidity  inland anglers | Standard error | Avidity Baltic Sea anglers | Standard error | Avidity North Sea anglers | Standard error |
| --- | --- | --- | --- | --- | --- | --- |
| No angling | 17.6 | 5.6 | 52.3 | 5.7 | 54.1 | 14.5 |
| Less than usual | 29.7 | 3.7 | 52.0 | 9.4 | 73.7 | 37.4 |
| Rather less than usual | 32.7 | 2.6 | 40.7 | 4.7 | 29.2 | 7.1 |
| The same as usual | 40.0 | 2.2 | 43.9 | 3.7 | 40.4 | 9.5 |
| Rather more than usual | 50.2 | 4.8 | 33.5 | 7.4 | 45.9 | 19.4 |
| More than usual | 63.1 | 6.8 | 39.1 | 11.6 | 104.3 | 65.6 |
| Design based KW test: | *t* = 68.2 d.f. = 5,  *p* < 0.0001 |  | *t* = 7.6  d.f. = 5,  *p* = 0.180 |  | *t* = 2.3  d.f. = 5,  *p* = 0.8 |  |

Table SR2.5 Self-assessed skill levels and angling activity of German anglers during the COVID-19 restriction period (March to May 2020) in weighted frequencies and proportions.

| Angling |  | Novice | Below average | Average | Above average | Expert |
| --- | --- | --- | --- | --- | --- | --- |
| No angling because of restrictions | Frequency | 38.2 | 31.9 | 121.9 | 86.8 | 42.1 |
| Angling during restriction |  | 47.1 | 106.4 | 446.5 | 477.1 | 276.1 |
| No angling because of restrictions | Proportion | 11.9 | 9.9 | 38.0 | 27.1 | 13.1 |
| Angling during restriction |  | 3.5 | 7.9 | 33.0 | 35.3 | 20.4 |

Table SR2.6 Probability table of skill rating and angling frequency in German inland waters, the Baltic Sea and the North Sea during the COVID-19 restriction period (March to May 2020) as stated by participants in the CATI survey (weighted percentage).

|  | Skill rating |  |  |  |  |
| --- | --- | --- | --- | --- | --- |
| Angling in inland waters | Novice | Below average | Average | Above average | Expert |
| No angling | 0.10 | 0 | 0.30 | 0.11 | 0.07 |
| Less than usual | 0.54 | 0.77 | 3.60 | 4.31 | 1.73 |
| Rather less than usual | 0.42 | 1.43 | 5.56 | 5.45 | 2.49 |
| The same as usual | 1.73 | 3.95 | 17.21 | 17.65 | 11.33 |
| Rather more than usual | 0.59 | 1.37 | 4.60 | 5.08 | 3.05 |
| More than usual | 0 | 0.26 | 1.64 | 2.72 | 1.97 |
| Angling in Baltic Sea | |  |  |  |  |
| No angling | 0 | 0.37 | 3.57 | 8.50 | 5.00 |
| Less than usual | 0.09 | 0.30 | 6.40 | 7.18 | 4.14 |
| Rather less than usual | 0.67 | 0.60 | 3.72 | 6.92 | 3.86 |
| The same as usual | 1.34 | 2.80 | 11.36 | 15.89 | 10.52 |
| Rather more than usual | 0.12 | 0.00 | 0.99 | 1.59 | 1.47 |
| More than usual | 0 | 0.35 | 0 | 1.99 | 0.27 |
| Angling in North Sea |  |  |  |  |  |
| No angling | 0 | 0 | 6.73 | 12.80 | 4.25 |
| Less than usual | 0 | 0.79 | 5.92 | 14.38 | 0.95 |
| Rather less than usual | 0 | 2.07 | 3.22 | 4.47 | 0.51 |
| The same as usual | 0 | 3.61 | 7.74 | 11.29 | 12.37 |
| Rather more than usual | 0 | 0 | 1.37 | 0.89 | 2.97 |
| More than usual | 0 | 0 | 2.97 | 0 | 0.69 |


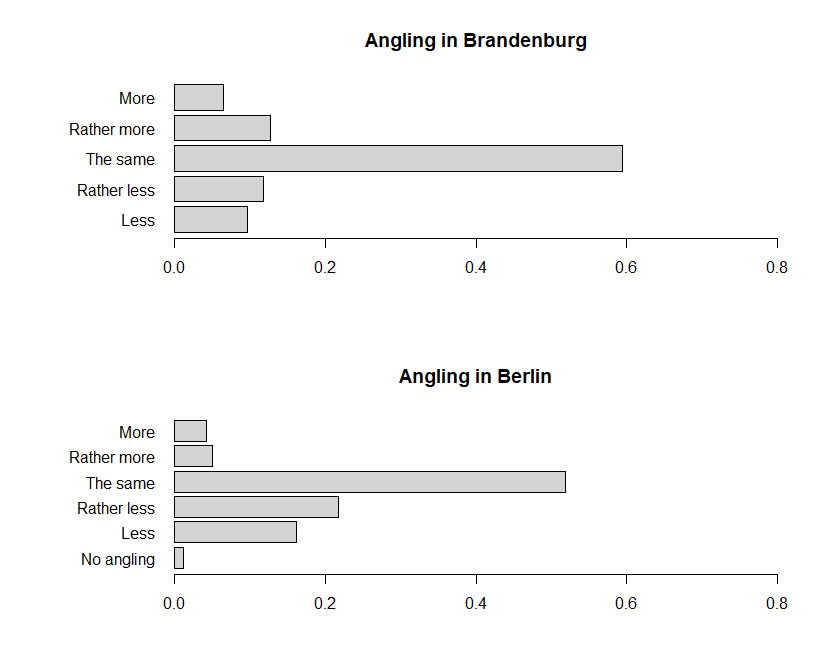


Figure SR2.1 Angling frequencies in inland waters during the COVID-19 restriction period (March to May 2020) in the two German federal states Brandenburg (rural) and Berlin (urban) (angler numbers in weighted frequencies and proportions).


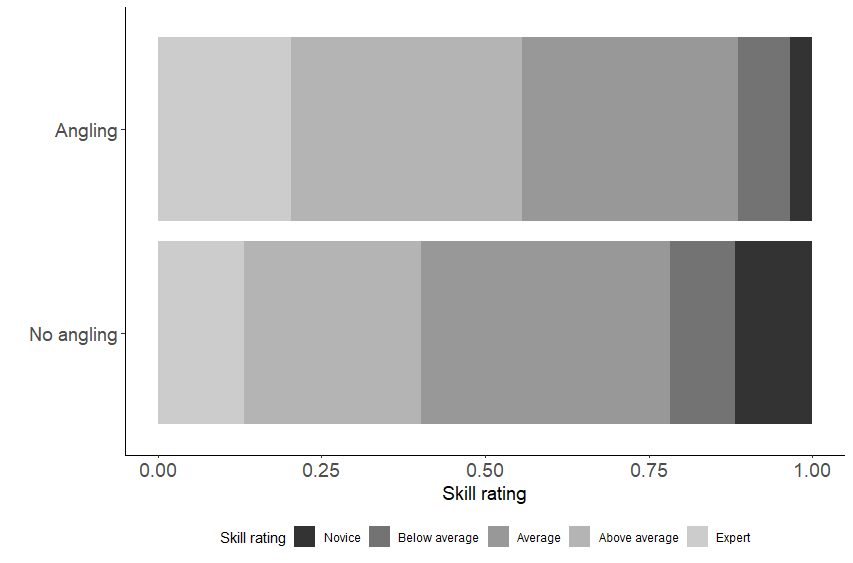


Figure SR2.2 Relative frequencies of the ratings of self-assessed angling skills by the participating anglers grouped by angling activity during the COVID-19 restriction period (March to May 2020).

**Supplementary material - Tables**

Table S1. Number of annual angling licence sales in Ontario, Canada, 2012 to 2020, according to angler origin (Canadian = angler with a non-Ontario address in Canada, Ontario residents, and Non-residents (generally anglers from the United States of America)

| Angler origin | 2012 | 2013 | 2014 | 2015 | 2016 | 2017 | 2018 | 2019 | 2020 |
| --- | --- | --- | --- | --- | --- | --- | --- | --- | --- |
| Ontario | 683,491 | 680,111 | 661,659 | 663,032 | 648,164 | 636,751 | 635,253 | 621,598 | 685,008 |
| Canada | 50,805 | 50,278 | 50,733 | 52,052 | 51,534 | 51,228 | 51,742 | 51,627 | 45,177 |
| Non-resident | 311,790 | 302,586 | 296,946 | 311,508 | 316,417 | 318,419 | 323,443 | 321,046 | 34,784 |

**Supplementary material - Figures**

**
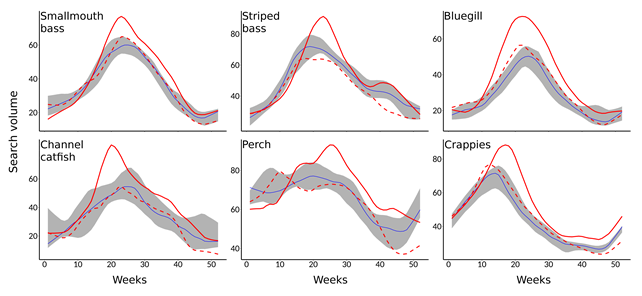
**Figure S1. Time series of relative search volumes for smallmouth bass, striped bass, bluegill, channel catfish, perch and crappies in the United States, based on Google Trends data. Full and dashed red lines represent weekly values for 2020 and 2021 respectively, while blue lines and grey shading represent median values and the range of values for the years 2017-2019 respectively Data were fitted with LOESS smoothing (*f* = 0.2). Please notice the different scales of the y-axes.

(a)


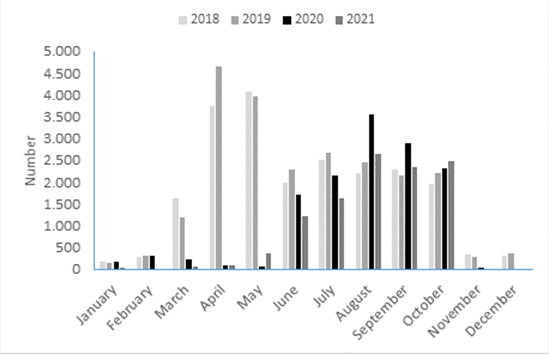


(b)


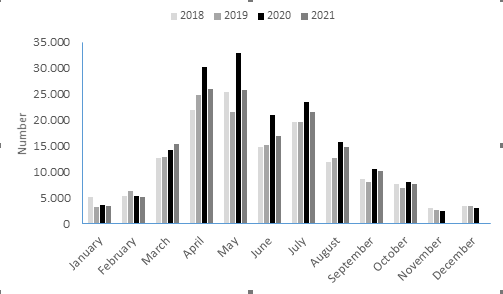


Figure S2. Fishing licence sales per month in Denmark for 2018 to 2021 for (a) weekly licences; and (b) annual licences.


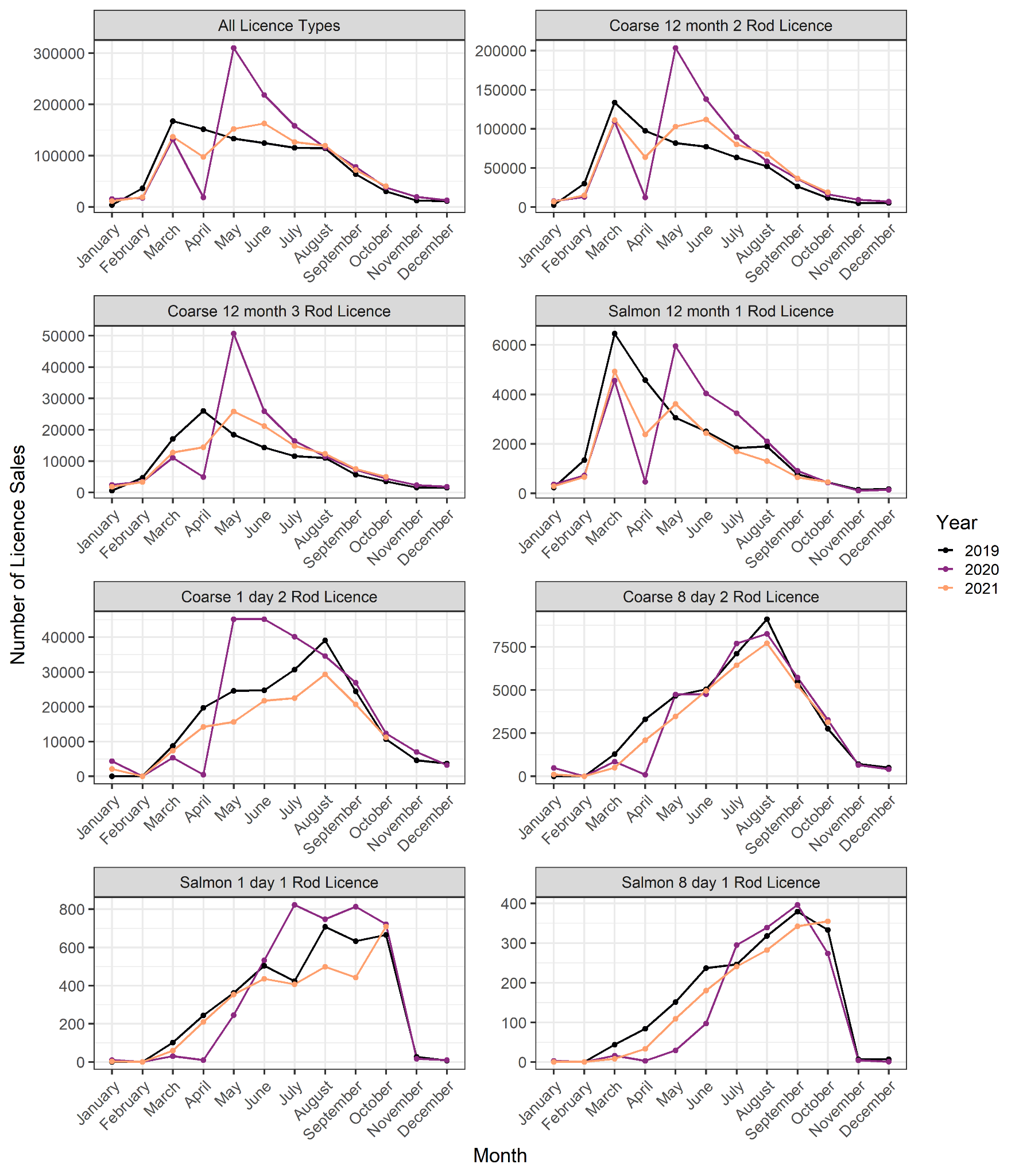


Figure S3. Fishing licence sales in England in 2019 (pre-pandemic lockdown), 2020 (with a lockdown period between March and May when angling was not permitted) and 2021 (where angling was allowed during the only lockdown period). Please notice the different scales of the y-axes.


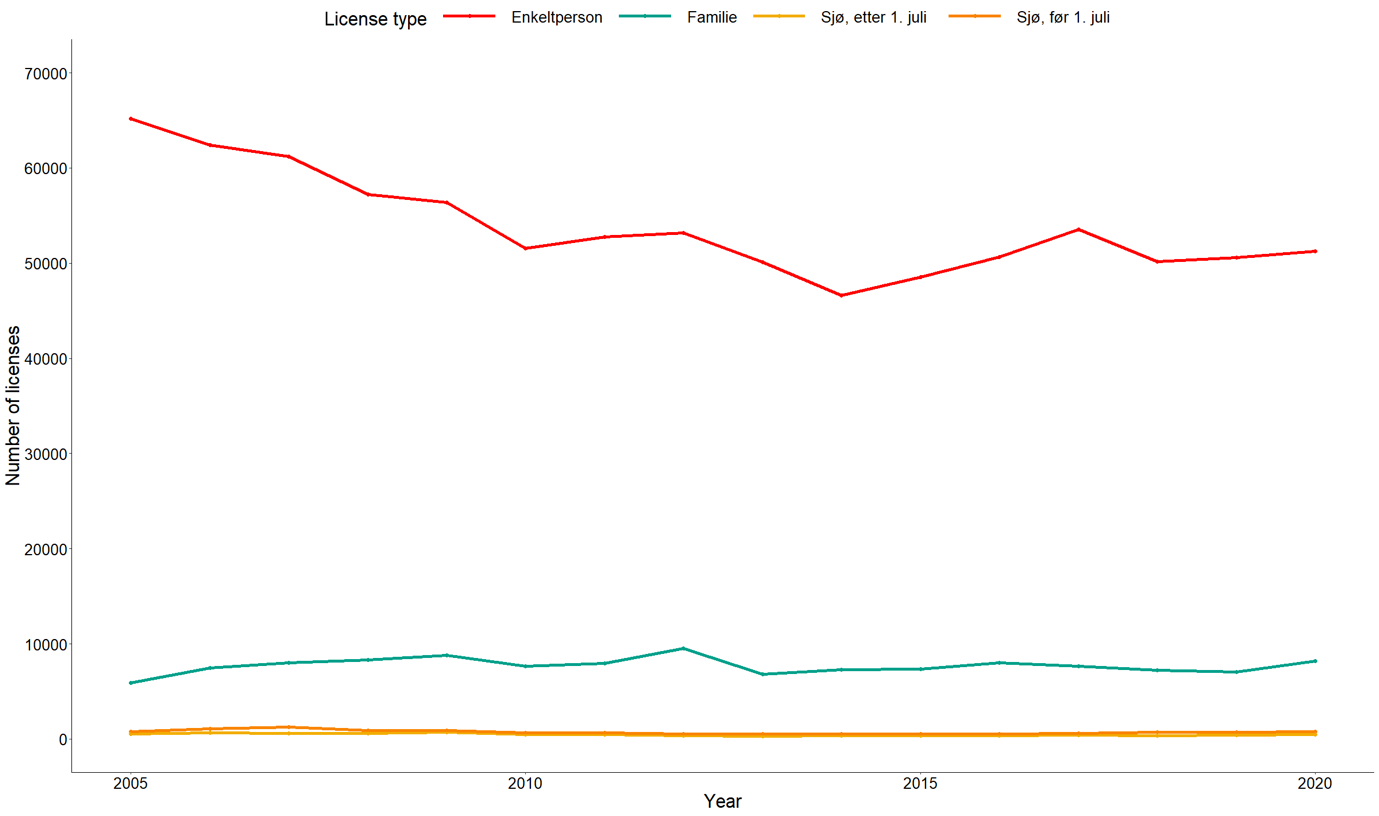


Figure S4. Annual fishing licence sales by category and by year for Norway from 2005 to 2020 (where red line: individual river licence; green: family river licence; yellow: sea licence after 1^st^ July, orange: sea licence before 1^st^ July.


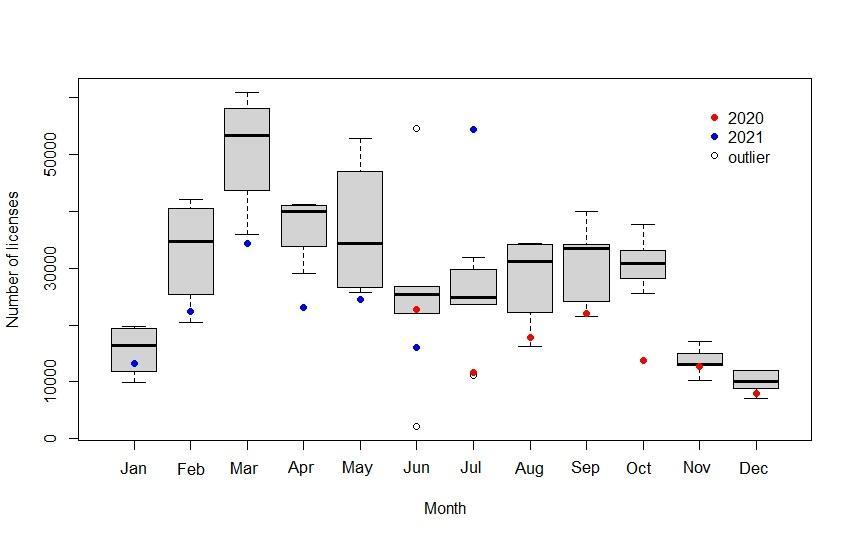


Figure S5. Box plot of recreational fishing licence sales by month in Brazil between 2010 and 2014, with sales in 2020 and 2021 shown separately.


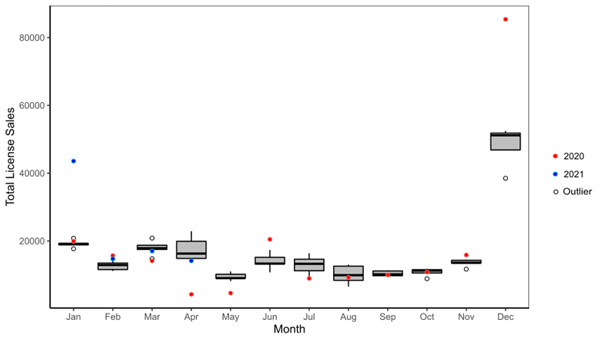


Figure S6. Monthly licence sales for the South African marine recreational fishery between 2015 and 2019 (box and whisker) and 2020 (red dots) and 2021 (blue dots).


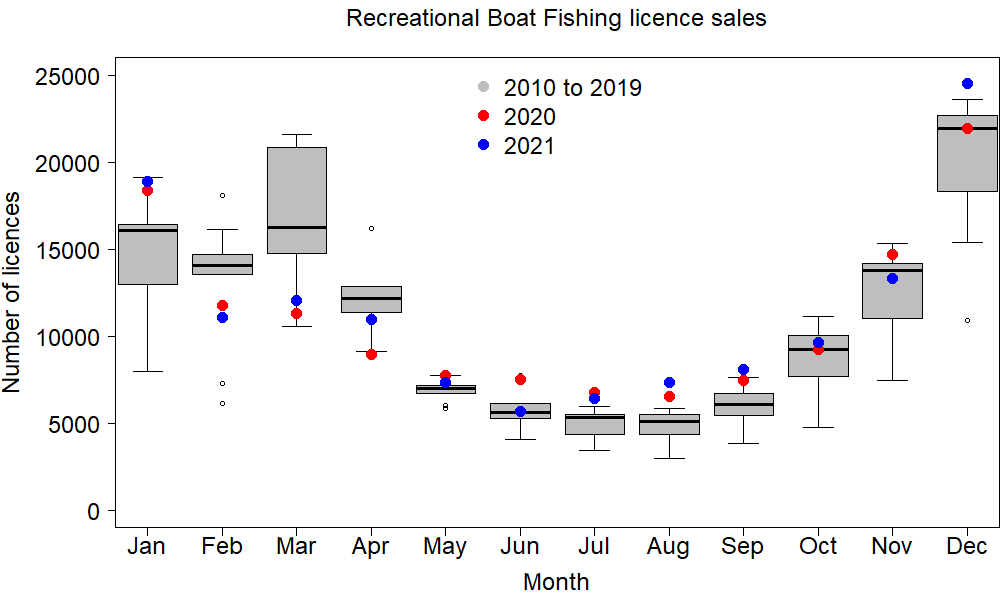


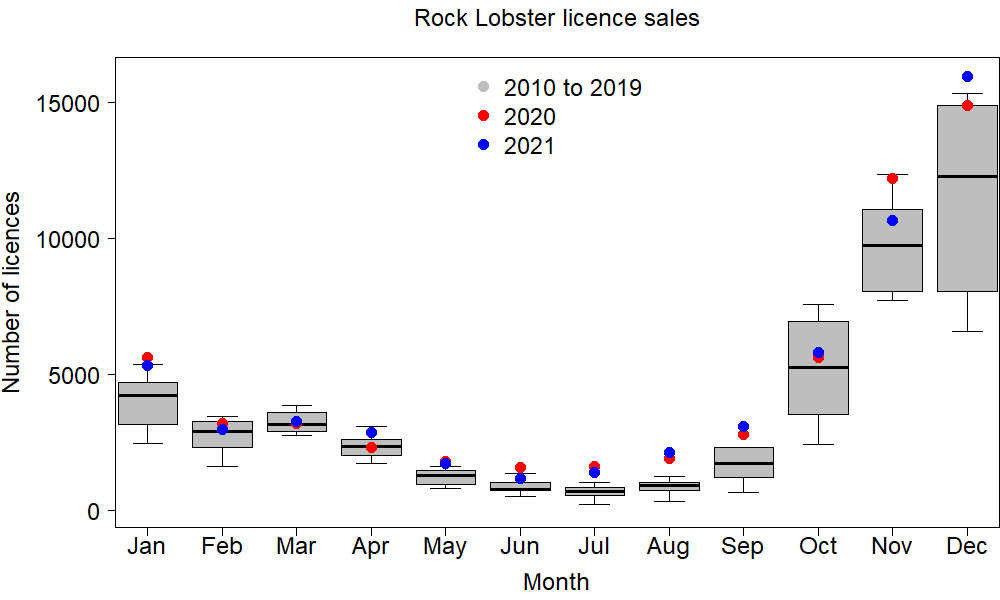


Figure S7. Box plot of recreational boat fishing and rock lobster recreational fishing licence sales by month in Western Australia between 2010 and 2019, with sales in 2020 and 2021 shown separately.


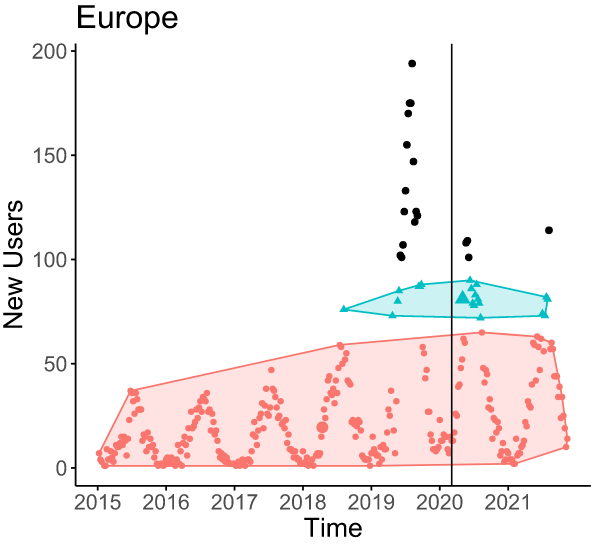

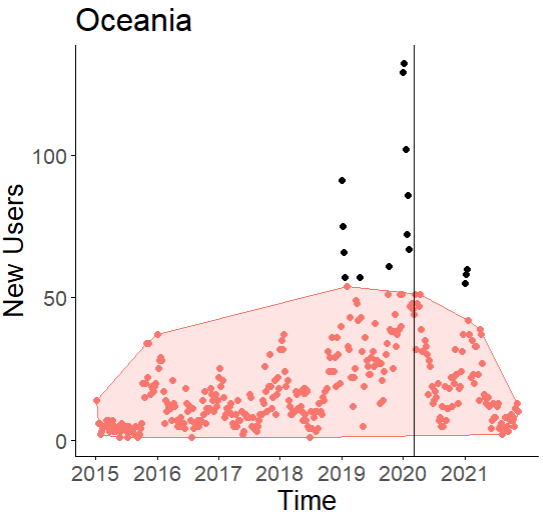

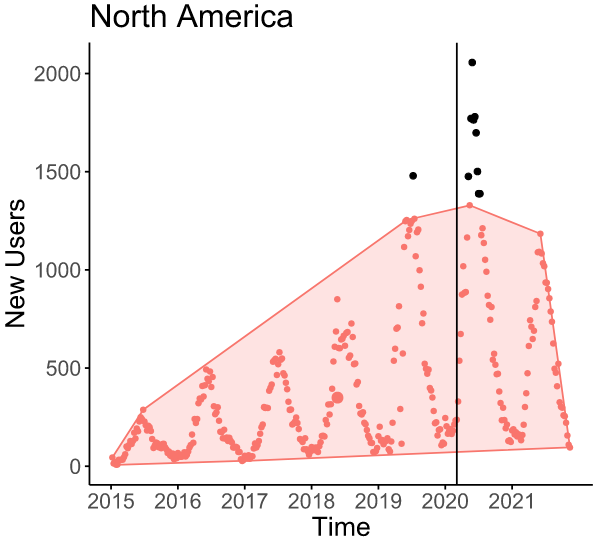


Figure S8. New users of the Fishbrain smartphone app each week by continent from 1 January 2015 to 31 October 2021. Shaded areas identify a collective pattern of the majority of data points, and black points are anomalies that are distinct from a pattern (see Section SM2 for details). The smaller cluster in the Europe panel identifies elevated recruitment in summer 2019 to 2021, but the outliers during this period show muted recruitment during the pandemic.


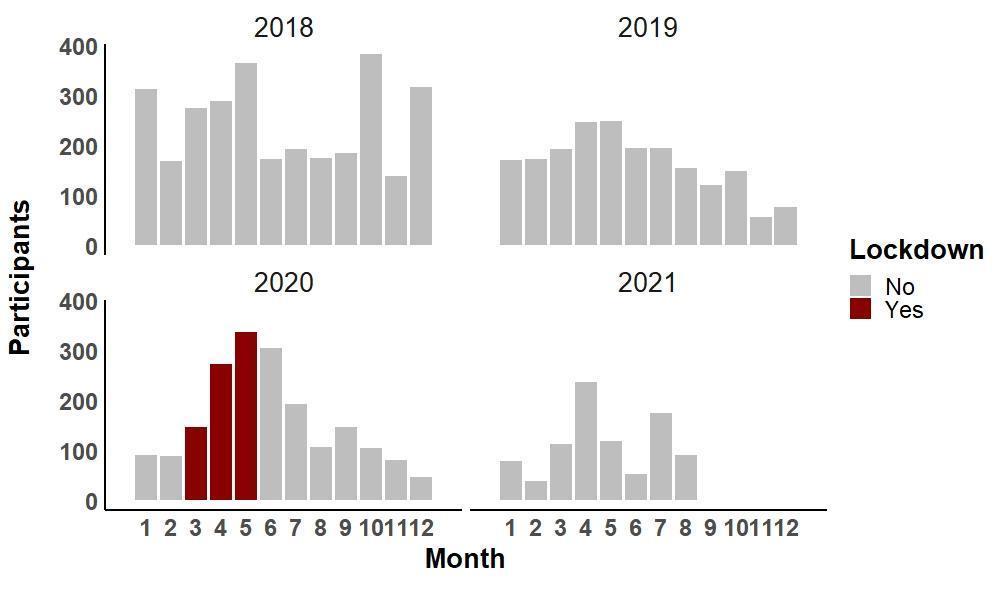


Figure S9. Number of new citizen science participants recruited to the Danish electronic citizen science platform ‘Fangstjournalen’ targeting recreational anglers during the lockdown period (March to May 2020).


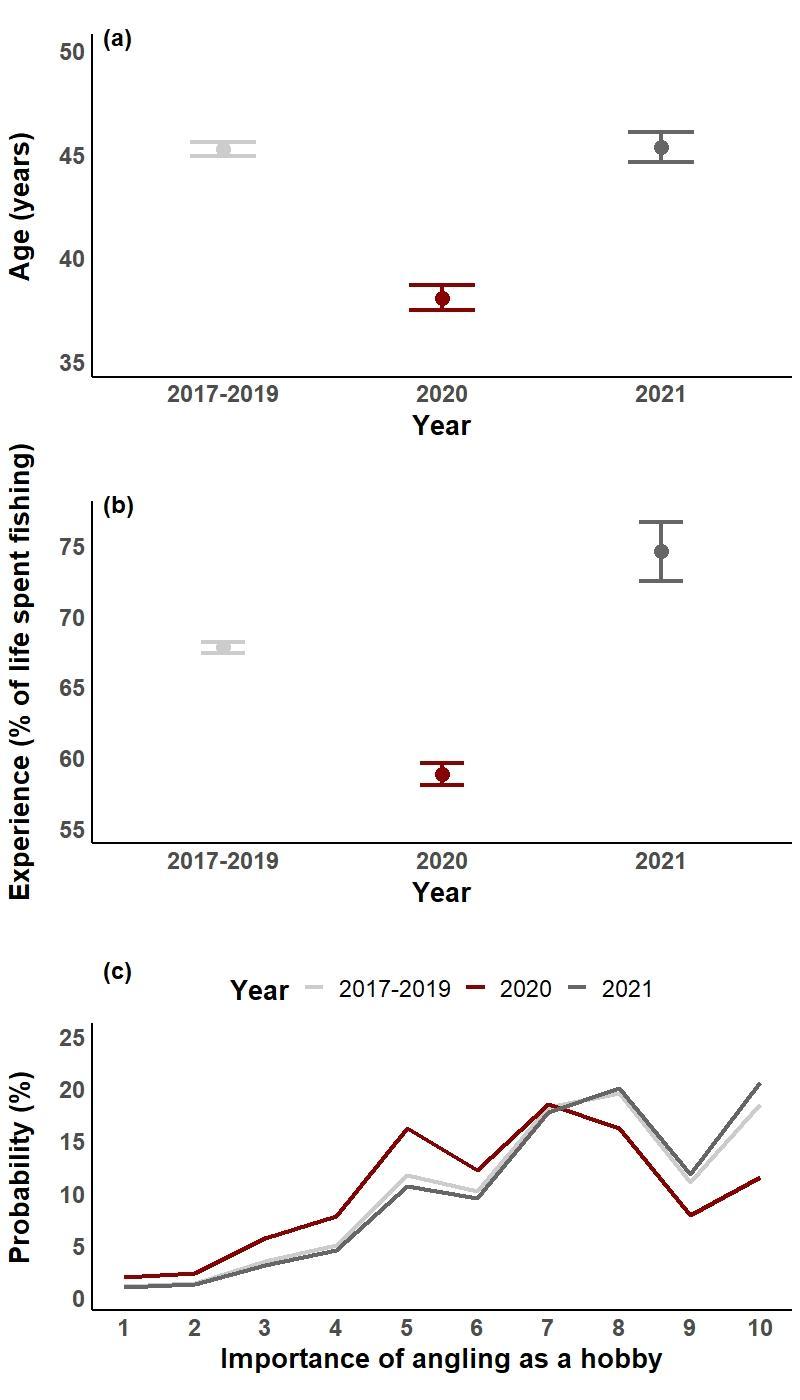


Figure S10. Analysis by age of (a) angler experience as proportion of life spent fishing (mean ± 95% CI) and (b) importance of angling as a hobby (Likert scale from 1 (low) to 10 (high)), shown as the average probability of a statement across the Likert scale; and (c) for anglers in Denmark recruited to the electronic citizen science platform in the period 11 March to 31 May during three different time periods (pre-pandemic (2017-2019 combined) and acute pandemic periods (lockdown periods) in 2020, and 2021). Data from the pre-lockdown period and the lockdown are equivalent to what is being presented by Gundelund and Skov (2021); 2021 data are unpublished.

**Acknowledgements**

We thank Derek Landry from the Ontario Ministry of Natural Resources and Forestry for furnishing us with data on fishing licence sales in Ontario. We also thank Jens Felix (Angler Association of Saxony, Germany), Sebastian Hanfland (State Fisheries Association of Bavaria, Germany), Marcel Weihenhahn (State Angler Association of Brandenburg, Germany), Thomas Richter und Thomas Schaarschmidt (LALFF Mecklenburg-Western Pomerania, Germany) for providing data on licence sales and memberships and Dieter Kömle for additional help. We thank the South African Department of Environment, Forestry and Fisheries for providing the marine angling licence information and Joel Shirlow (Department of Primary Industries and Regional Development) for access to monthly licence information of Western Australia. We thank Fishbrain for access to their global dataset, and Stig Prüssing (The Danish Fishery Agency) for access to the Danish fishing licence sales. Thanks to the Environment Agency for provision of licence sales data for England, and to the Departamento de Registro e Monitoramento de Aquicultura e Pesca (Secretaria de Aquicultura e Pesca) for providing more recent data on licence purchases for Brazil. CS and CG thank the citizen scientists who provided data for the Danish citizen science platform Fangstjournalen. CG and CS received funding from the Danish Rod and Net Fish Licence funds (project 39122). CS, MSW and the German telephone survey have been cofounded by the European Commission’s Data Collection Framework (DCF). WCL and HVS received ﬁnancial support from the Federal Ministry of Education and Research of Germany in the framework of marEEshift (project no. 01LC1826B). WMP and KH were supported by the One Ocean Hub, an independent programme for collaborative research for development, funded by UK Research and Innovation (UKRI) through the Global Challenges Research Fund (GCRF).

All authors contributed to the design of the work, the analysis and interpretation of data, all authors contributed to the drafting of the manuscript and approved the version that was submitted. The views expressed are those of the authors, not their parent organisations, but are supported by the U.S. Geological Survey. Any use of trade, firm, or product names is for descriptive purposes only and does not imply endorsement by the US Government.

**References**

Ester, M., Kriegel, H.P., Sander, J. and Xu, X., 1996, August. A density-based algorithm for discovering clusters in large spatial databases with noise. In *kdd* (Vol. 96, No. 34, pp. 226-231).

Hahsler, M., Piekenbrock, M. and Doran, D., 2019. dbscan: Fast density-based clustering with R. Journal of Statistical Software, 91(1), 1-30.

Lewin, W.-C.**,** Weltersbach, M.S.**,** Haase, K., Strehlow, H.V., 2021. Who travels how far: German Baltic sea anglers’ travel distances as precondition for fisheries management and coastal spatial planning. *Ocean Coast. Manag.* 209, 105640. https://doi.org/10.1016/j.ocecoaman.2021.105640.

Lumley, T., 2020. Survey: analysis of complex survey samples. R package version 4.0.).

R Core Team, 2020. R: a language and environment for statistical computing. R Foundation for Statistical Computing, Vienna, Austria.

R Core Team, 2021. R: a language and environment for statistical computing. R Foundation for Statistical Computing, Vienna, Austria.

Strehlow, H.V., Schultz, N., Zimmermann, C., Hammer, C., 2012. Cod catches taken by the German recreational fishery in the western Baltic Sea, 2005-2010: implications for stock assessment and management. ICES J. Mar. Sci., 69, 1769–1780.

Van Gemert, R., Koemle, D., Winkler, H., Arlinghaus, R., 2022. Data‐poor stock assessment of fish stocks co‐exploited by commercial and recreational fisheries: Applications to pike Esox lucius in the western Baltic Sea. *Fisheries Management and Ecology*, *29*(1), 16-28.
